# Epigenetics of Mitochondria-Associated Genes in Striated Muscle

**DOI:** 10.3390/epigenomes6010001

**Published:** 2021-12-22

**Authors:** Kenneth C. Ehrlich, Hong-Wen Deng, Melanie Ehrlich

**Affiliations:** 1 Center for Bioinformatics and Genomics, Tulane University Health Sciences Center, New Orleans, LA 70112, USA; kehrlich@tulane.edu (K.C.E.); hdeng2@tulane.edu (H.-W.D.); 2 Tulane Cancer Center and Hayward Genetics Center, Tulane University Health Sciences Center, New Orleans, LA 70112, USA

**Keywords:** skeletal muscle, heart, enhancer, epigenetics, DNA methylation, mitochondria, mitophagy, Parkinson’s disease, PGC-1α/PPARGC1A, PRKN/PARK2

## Abstract

Striated muscle has especially large energy demands. We identified 97 genes preferentially expressed in skeletal muscle and heart, but not in aorta, and found significant enrichment for mitochondrial associations among them. We compared the epigenomic and transcriptomic profiles of the 27 genes associated with striated muscle and mitochondria. Many showed strong correlations between their tissue-specific transcription levels, and their tissue-specific promoter, enhancer, or open chromatin as well as their DNA hypomethylation. Their striated muscle-specific enhancer chromatin was inside, upstream, or downstream of the gene, throughout much of the gene as a super-enhancer (*CKMT2*, *SLC25A4*, and *ACO2*), or even overlapping a neighboring gene (*COX6A2*, *COX7A1*, and *COQ10A*). Surprisingly, the 3′ end of the 1.38 Mb *PRKN* (*PARK2*) gene (involved in mitophagy and linked to juvenile Parkinson’s disease) displayed skeletal muscle/myoblast-specific enhancer chromatin, a myoblast-specific antisense RNA, as well as brain-specific enhancer chromatin. We also found novel tissue-specific RNAs in brain and embryonic stem cells within *PPARGC1A* (*PGC-1α*), which encodes a master transcriptional coregulator for mitochondrial formation and metabolism. The tissue specificity of this gene’s four alternative promoters, including a muscle-associated promoter, correlated with nearby enhancer chromatin and open chromatin. Our in-depth epigenetic examination of these genes revealed previously undescribed tissue-specific enhancer chromatin, intragenic promoters, regions of DNA hypomethylation, and intragenic noncoding RNAs that give new insights into transcription control for this medically important set of genes.

## 1. Introduction

Striated muscle consists of skeletal muscle (SkM), the largest organ in the body, and heart muscle, whose malfunction is responsible for approximately 30% of deaths globally [1]. This type of muscle is characterized by repeated highly organized units, which are called sarcomeres, and contain myosin and actin filaments that allow muscle to contract [2]. The sarcomere banding pattern in striated muscle fibers is absent from the other type of muscle, smooth muscle, e.g., the muscle in aorta [3]. A major difference between human cardiac muscle and SkM is that mature SkM cells are very highly multinucleated with hundreds of nuclei in giant cells while the muscle-specific cells in heart (cardiomyocytes) are predominantly mononuclear or binuclear and normally much smaller than SkM cells [2,4,5]. Although cardiomyocytes are not highly polyploid, they form an electrical syncytium to facilitate coordinated heart contractions, unlike SkM cells, which are electrically isolated from each other [2].

The progenitor cells for SkM formation and repair are myoblasts, which, upon differentiation, can develop into multinucleated myotubes or fuse with multinucleated muscle fibers [6]. Postnatally, myoblasts are a transient cell type that is generated upon induction of differentiation of adult muscle stem cells called satellite cells [5]. During embryogenesis, cardiomyocytes are formed from precursor cells, and heart is the first organ to develop into a recognizable and functional organ [7]. Multiple types of cardiac progenitor cells from different developmental lineages are involved in heart formation [8] and these lineages diverge according to the chamber of the heart [9]. Early embryonic cells that are multipotent in the cardiopharyngeal field can differentiate into committed cardiac progenitor cells or SkM cells of the head or neck [9]. In addition to the cells in the muscle lineages of SkM and heart, both organs contain cells from other lineages, such as fibroblasts [4,10]. Moreover, morphological and physiological differences in heart chambers and in SkM type (and fiber type [11,12]) are accompanied by differences in transcription profiling, although cardiac muscle forms one interrelated group distinct from several SkM subgroups [3].

Most of the energy required for muscle contraction comes from oxidative phosphorylation rather than from glycolysis [12]. Cardiac muscle and, to a lesser extent, fast oxidative muscle fibers are particularly rich in mitochondria because of the large amounts of ATP required continuously by heart and during exercise by SkM [13,14]. The mitochondrion is a dynamic double-membraned organelle enabling ATP production and transport. It also contributes to apoptosis, autophagy, production of reactive oxygen species (ROS), calcium handling, and cell fate decisions in stem cells [11,13]. Besides having a critical role in differentiation, physiological changes, and response to stress, mitochondrial dysfunction contributes to aging and to many diseases, including inherited myopathies, cardiac dysfunction, and cancer [13,15,16,17,18]. Although the mitochondrion is self-replicating and possesses its own genome, most proteins in mitochondria are encoded by the nucleus [15]. The master regulator of mitochondrial biogenesis is the nuclear-encoded transcription coregulator peroxisome proliferator-activated receptor coactivator 1A (PPARGC1A/PGC-1α) [19]. Balancing mitochondrial biogenesis is the selective autophagy of mitochondria, called mitophagy. Mitophagy removes dysfunctional mitochondria and thereby helps to maintain the capacity to synthesize sufficient ATP, to generate needed levels of intermediate metabolites, and to decrease levels of reactive oxygen species (ROS) [20].

Given the central role of epigenetics in differentiation, homeostasis, and disease, in general, and to striated muscle in particular [21,22,23,24,25], we studied the epigenetics of genes associated with both types of striated muscle. We first identified a set of genes that are preferentially expressed in SkM and heart at similar levels (but not in aorta) and then determined which functional groups are most overrepresented in this set of genes. We found that nuclear genes encoding proteins resident in mitochondria or controlling mitochondrial biogenesis or degradation were the most abundant subgroup of SkM- and heart-preferentially transcribed genes. Our study of the relationship of the epigenomics and transcriptomics of these genes and their gene neighborhoods using profiles from the ENCODE and Roadmap Epigenomics Projects [26,27] provides new insights into the control of striated muscle gene transcription by a variety of types of epigenetic regulation at promoters, intragenic and intergenic enhancers, and enhancers in neighboring genes.

## 2. Results

### 2.1. Gene Selection Strategy

**Figure 1:**
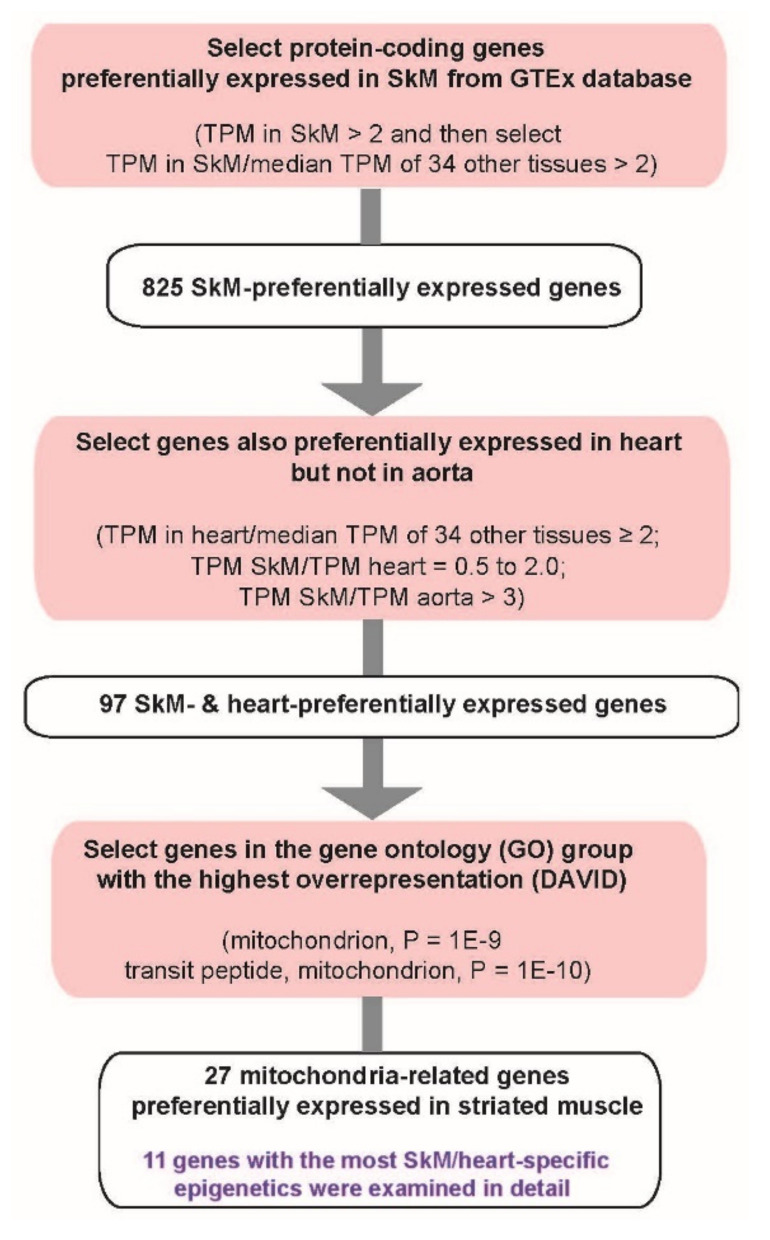
Flow scheme showing the strategy for selection of genes preferentially expressed in SkM and heart and associated with mitochondrial function, structure, biogenesis, or mitophagy.

We identified protein-coding nuclear genes that are preferentially expressed in SkM and cardiac muscle, but not in smooth muscle, using the GTEx poly(A)^+^ RNA-seq database [28] that includes hundreds of replicate samples for each of dozens of human tissues. First, we obtained all the protein-coding genes with TPM > 2 in SkM and then selected for those with ratios of TPM in SkM (gastrocnemius muscle) to the median TPM for heterologous tissues (34 types) > 2 (Figure 1). From the 825 genes that met this criterion, we retained only genes that also exhibited a ratio of TPM in heart (left ventricle) to the median TPM for heterologous tissues ≥ 2 and a ratio of TPM in SkM to TPM in heart (left ventricle) between 0.5 and 2.0. When we used the GTEx RNA-seq data for right atrial appendage, the only other heart tissue in this database, similar ratios of expression in SkM to heart (atrial appendage) were obtained (Table S1; *MYL2* was the sole exception). Our last transcription criterion was to require a ratio of TPM in SkM to TPM in aorta (a non-striated muscle-rich tissue [3]) >3, which gave a total of 97 genes (Table S1) that are highly expressed in SkM and heart at similar levels with much lower expression in aorta and most other tissues.

Next, we determined which categories of genes (gene ontology, GO, or other functional gene groups) are overrepresented among the 97 genes. GO analysis using DAVID (Database for Annotation, Visualization and Integrated Discovery v6.8; Table S2) showed the strongest enrichment for the terms mitochondrion (P = 1 × 10^−9^; 27 genes) and transit peptide: mitochondrion (P = 1 × 10^−10^; 18 genes most of which were included in the “mitochondrion” GO term) consistent with a previous transcriptomic and proteomic study of striated muscle [29]. We excluded three genes, *AGAP2*, *MYOM1*, and *TNNC1*, from further consideration because, they are only weakly associated with mitochondrial function. The remaining genes in these two gene sets were combined (Table 1). Eleven of the 27 genes that had the strongest correlations between preferential expression in striated muscle and their tissue-specific epigenetics were examined in detail. As expected, after mitochondrial categories, the next most overrepresented GO terms for genes preferentially expressed in SkM and heart were “muscle protein” and “M band” (Table S2), but genes in those categories were not included in this study.

### 2.2. COX6A2 and COX7A1, Highly and Preferentially Expressed Genes in SkM and Heart, Are Embedded in Promoter Chromatin Surrounded by Enhancer Chromatin in These Tissues

**Figure 2:**
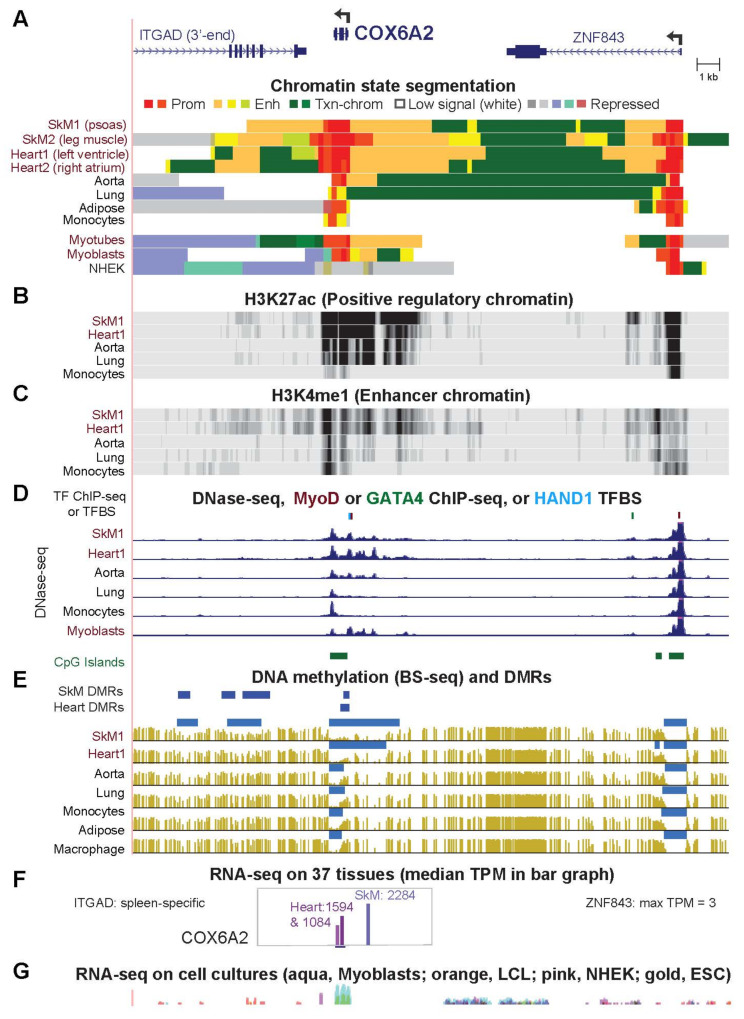
*COX6A2* displays striated muscle-specific epigenetic marks including in its gene neighbors. (**A**) *COX6A2* gene neighborhood (chr16:31,430,114–31,456,585) with chromatin state segmentation profiles (18-state; Roadmap Epigenomics [27]; Prom, promoter chromatin; Enh, enhancer chromatin, Txn-chrom, H3K36me3-enriched chromatin; SkM1, psoas muscle; SkM2, upper leg muscle; Heart1, left ventricle; Heart2, right atrium; NHEK, embryonic kidney cells; ESC, embryonic stem cells (H1); LCL, lymphoblastoid cell line (GM12878). (**B**) H3K27ac and (**C**) H3K4me1 signal profiles. (**D**) Transcription factor (TF) ChIP-seq and DNase-seq; tick marks, sites bound by SkM-associated MyoD in myoblasts or heart-associated GATA4 in iPSC-derived cardiomyocytes that overlapped DNaseI hypersensitive sites (DHS) in SkM or heart, respectively. HAND1 TFBS, strongly predicted binding sites for HAND1, which lacks ChIP-seq data. (**E**) Bisulfite-seq (BS-seq) and SkM and heart DMRs (vs. aorta, lung, monocytes, adipose, and macrophage). Dark blue bars, hypomethylated DMRs; light blue bars, regions of significantly lower methylation relative to the whole genome for the same tissue. (**F**) RNA-seq bar graph for 36 tissues showing median TPM in linear scale [28]; horizontal black line under the graph, the region evaluated. (**G**) Cell culture RNA-seq [26] shown as an overlay of the indicated four cell types (log scale). All tracks are horizontally aligned in this and subsequent figures and are from the UCSC Genome Browser [32].

*COX6A2* and *COX7A1* are very small genes (0.6 and 1.5 kb, respectively) that encode subunits of the mitochondrial cytochrome c oxidase [30] and exhibit the highest expression in SkM of all the 26 studied striated muscle/mitochondria-associated genes (Table 1). Their expression in heart is only a little less than in SkM (*COX6A2* TPM: SkM, 2284 and heart, 1593; *COX7A1* TPM: SkM, 704 and heart 584). *COX6A2* has the highest specificity for SkM of all the striated muscle/mitochondria-associated genes. The specificity of *COX7A1* for striated muscle is also high but lower than that of *COX6A2*, which is reflected in the epigenetic profiles of these two genes (Figure 2 and Figure S1). Two of the eleven other genes encoding subunits of cytochrome c oxidase (*COX5A* and *COX10*) were preferentially expressed in SkM and heart but with less specific transcription and epigenetics than for *COX6A2* and *COX7A1* (Table 1) [31].

Only SkM (both psoas and leg muscle) and heart (both left ventricle and right atrium) had promoter chromatin (red segments) throughout the *COX6A2* gene body and strong enhancer chromatin nearby (orange or yellow-green segments; Figure 2A). Relative to the other tissues, SkM and heart had the strongest enhancer-determining histone H3 lysine 27 acetylation (H3K27ac) plus H3 lysine 4 monomethylation (H3K4me1) upstream and downstream of the gene (Figure 2B,C). Remarkably, the downstream enhancer chromatin in striated muscle extends into *ITGAD*, an integrin subunit-encoding gene with negligible expression in tissues other than spleen (Table S3). In addition, *ZNF843*, the uncharacterized upstream gene neighbor of *COX6A2*, harbored enhancer chromatin in intron 1 specifically in SkM and heart, although this gene, like *ITGAD*, displayed negligible RNA levels in these two tissues (Table S3). In the 1-Mb neighborhood of *COX6A2*, only one other gene, *TRIM72*, which plays a major role in cell membrane repair [30], displays specific expression in SkM (data not shown). Upstream of *COX6A2*, the most DNaseI hypersensitivity was seen in SkM, heart, myoblasts, and myotubes (Figure 2D and data not shown).

*COX6A2* in aorta (TPM, 4) had more DNaseI hypersensitivity and enhancer chromatin upstream of the gene than did most other non-striated muscle tissues (median TPM, 1) but less than in SkM and heart. Aorta also differed from striated muscle in its DNA methylation profile in this gene region. We used two quantitative measures of differential DNA methylation. One of these shows regions that had significantly lower methylation than in the same genome as a whole [33], which we refer to as low methylated regions (LMRs, horizontal light blue bars over individual bisulfite-seq profiles, Figure 2E). The other is statistically significant, hypomethylated differentially methylated regions (DMRs, dark blue bars above all the bisulfite-seq tracks) determined by comparing bisulfite-seq profiles of SkM to those of lung, aorta, adipose, monocytes, and heart (SkM DMRs) or heart to lung, aorta, adipose, monocytes, and SkM (heart DMRs) [22]. *COX6A2* had negligible levels of DNA methylation over much of the CpG-rich region (CpG island) that covered this small gene in all tissues. However, there was significantly less DNA methylation at the 5′ end of the gene and immediately upstream in SkM and heart compared with aorta and other tissues (Figure 2E). In addition, SkM-specific hypomethylated DMRs were observed downstream of *COX6A2* overlapping the 3′ end of *ITGAD* (Figure 2E).

*COX7A1*, unlike *COX6A2*, displayed enhancer chromatin bordering promoter chromatin over the gene in many tissues (Figure 2A and Figure S1), consistent with the differences in the median TPM for expression of these genes in non-striated muscle tissues (56 and 1, respectively, Table 1). Larger tissue-specific differences in epigenetics of *COX7A1* were seen for DNA methylation (Figure S1). A CpG island that overlaps *COX7A1* displayed tissue-specific DNA methylation, unlike most CpG island promoters, with the least methylation in SkM, heart, and aorta (TPM, 704, 584 and 163, respectively). In addition, DNA hypomethylation in SkM and heart extended from upstream of *COX7A1* to the 3′ end of the downstream gene, *CAPNS1* (*Calpain Small Subunit 1*). *CAPNS1*, which encodes a subunit of cysteine proteinase, is broadly expressed with no specificity for SkM (Table S3). This suggests that the SkM/heart hypomethylated DMRs at its 3′ end favor transcription of *COX7A1*, and not *CAPNS1*.

*COX7A1* and *COX6A2* are also preferentially expressed in myoblasts and myotubes (Figure 2E,F and Figure S1; Table S4). However, myoblasts and myotubes lack the gene-downstream enhancer chromatin for both genes (Figure 2A,G and Figure S1). We looked at genome-wide profiles of binding of MyoD [34], a myogenesis-associated TF [6] that is involved in adult SkM function as well as in embryonic SkM formation and SkM regeneration [35]. The gene (*MYOD1*) encoding the specificity-determining subunit of MyoD has negligible expression in 52 examined adult tissues [28] other than SkM (TPM, 22) and testis (TPM, 1). Binding sites for MyoD in myoblasts were seen 0.1 kb upstream of the transcription start site (TSS) of *COX6A2* and of *COX7A1* (Figure 2D and Figure S1).

We looked for binding of the heart-associated TFs [9,36] GATA4 and TBX5 in iPSC-derived cardiomyocytes and for stringently predicted binding sites for NKX2-5 and HAND1 (for which heart-related ChIP-seq profiles were not available) at heart DHS near *COX6A2* and *COX7A1*. We chose these four cardiogenesis-associated TFs because their expression [28] is specific for postnatal heart (ratios of TPM in left ventricle or right atrial appendage/median TPM for 35 heterologous tissues were >100). DNA binding sites for SMARCD3 and MEF2C, TFs that are also important for cardiogenesis [7], were not examined because the genes that encode them lack preferential expression in postnatal heart (ratios of TPM for left ventricle or right atrial appendage/median TPM of 35 non-striated muscle tissues = 0.5–1.6). GATA4, TBX5, NKX2-5 and HAND1 are not only involved in embryonic heart morphogenesis but also in postnatal heart function [37,38,39]. We found evidence for HAND1 sites in the striated muscle-associated DHS within 0.2 kb of the *COX6A2* TSS (Figure 2D) and for a MyoD site and three HAND1 sites overlapping DHS near the TSS of *COX7A1* (Figure S1).

### 2.3. COQ10A, HADHB, and CPT1B Have Adjacent Mitochondria-Associated Genes That Share Enhancer or Promoter Chromatin with Them in Skeletal Muscle and Heart

**Figure 3:**
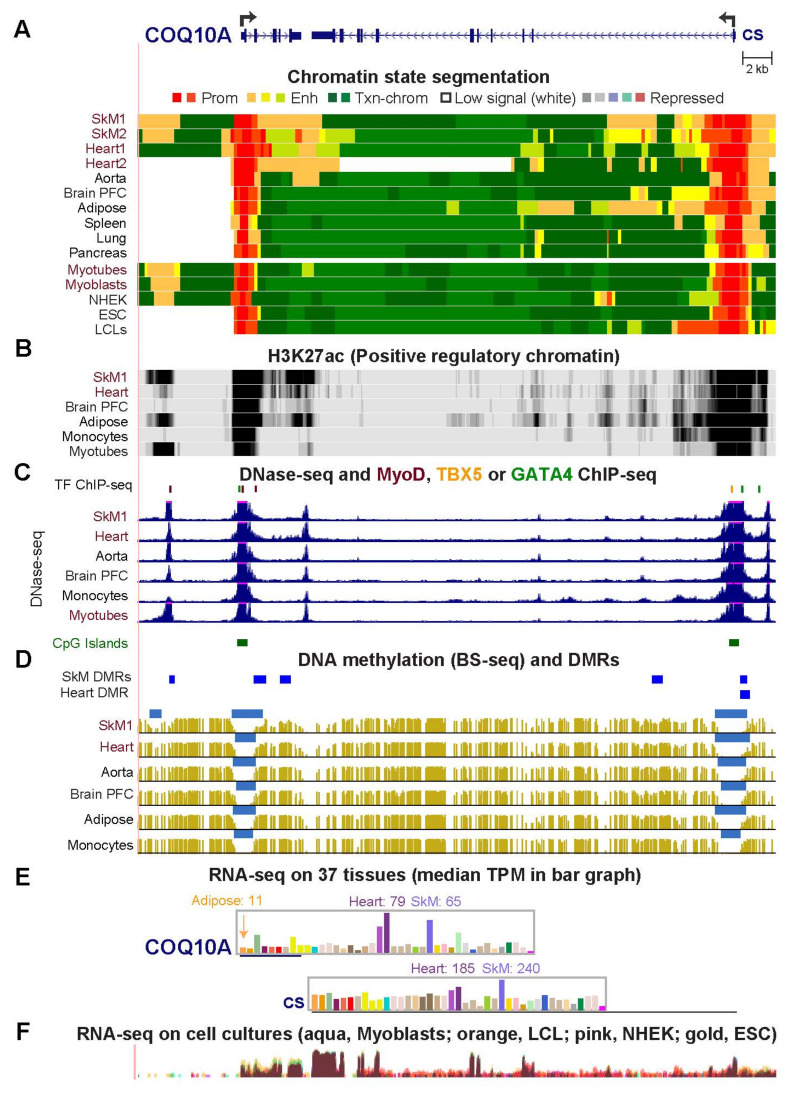
*COQ10A (Coenzyme Q10A)* and its neighbor, *CS (Citrate Synthase)* share epigenetic features consistent with their coordinated preferential expression in striated muscle. The region shown is chr12:56,653,687–56,696,821. (**A**–**F**), similar to panels in Figure 2 except that the H3K4me1 tracks are not shown, and panel (**C**) shows binding of TBX5 and GATA4 in iPSC-induced cardiomyocytes cells at DHS seen in heart. In panel (**E**), different linear scales are used for the two genes. The median TPM for heart is shown for the left ventricle (with the atrial appendage bar to its left) and, in the case of adipose tissue, for subcutaneous adipose (with the bar for visceral adipose tissue to its right). Brain PFC, prefrontal cortex.

*COQ10A* (*Coenzyme Q10A*; Figure 3) codes for a mitochondrial redox respiratory electron and proton transport protein [30] (Table S5). Its functionally related downstream neighbor *CS* (*Citrate Synthase*, Mitochondrial; ENSG00000062485) encodes a Krebs cycle enzyme [30]. The 3′ ends of *COQ10A* and *CS* are only 0.7 kb apart. Like *COQ10A*, *CS* is expressed at its highest levels in SkM and heart but with insufficient specificity to meet our criteria for preferential expression in striated muscle (Table S3). Strong enhancer chromatin segments (orange or yellow-green, Figure 3A) bordering promoter chromatin (red, Figure 3A) were observed for *COQ10* and *CS* but the enhancer chromatin was more specific for striated muscle in the case of *COQ10A*. Enhancer chromatin that spanned the adjacent 3′ ends of *COQ10A* and *CS* is much closer to the promoter of *COQ10A* than to the promoter of the *CS* gene. This might help explain the higher striated muscle specificity for expression of *COQ10A* relative to *CS*. SkM-associated peaks of H3K27ac signal, MyoD binding at two DHS, and SkM-hypomethylated DMRs near the *COQ10A* TSS may also contribute to the SkM-preferential expression of this gene (Figure 3B–E). In contrast to its SkM-preferential expression, *COQ10A* is not preferentially expressed in myoblasts vs. other cell cultures (Figure 3F). This could be due partly to the lower amounts of enhancer chromatin at the gene in myoblasts than in SkM. In cardiomyocytes, *COQ10A* and *CS* bound heart-related TFs GATA4 or TBX5 in a region overlapping a constitutive promoter DHS (Figure 3C).

A different type of sharing of epigenetic marks was observed for the bidirectional promoter of *HADHA* and *HADHB (*Figure S2). These genes encode subunits of a tetrameric hydroxyacyl-CoA mitochondrial dehydrogenase [30] and, therefore, their coregulation is clearly advantageous. Although both *HADHA* and *HADHB* have their highest expression in striated muscle, only *HADHB* met the expression ratio requirements for SkM-and-heart preferential expression (Table 1 and Table S3). Accordingly, strong enhancer chromatin and DNA hypomethylation in intron 1 was more specific for SkM and heart at *HADHB* than at *HADHA* (Figure S2).

**Figure 4:**
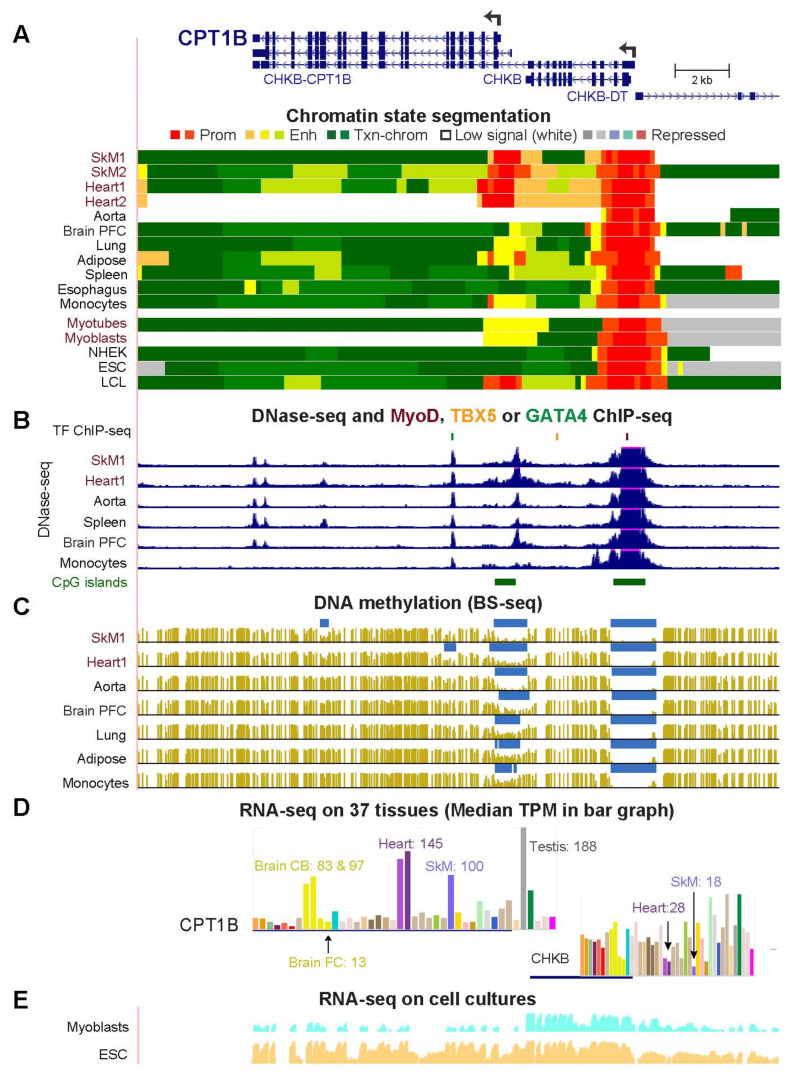
Upstream enhancer chromatin and promoter chromatin are associated with preferential expression in striated muscle of *CPT1B* but not its neighbor *CHKB*. The region shown is chr22:51,003,006–51,026,789. The 5′ end of *CHKB-DT* (lncRNA gene) is seen. (**A**–**E**), like Figure 3 except that the RNA-seq profiles for myoblasts and the ESC are shown separately. CB, cerebellum; PFC, prefrontal cortex.

Unlike the 3′ to 3′ or 5′ to 5′ juxtapositions of *COQ10A* with *CS* and *HADHB* with *HADHA*, the 5′ end of *CPT1B* (*Carnitine Palmitoyltransferase 1B*) is adjacent to the 3′ end of its broadly expressed neighbor *CHKB* (*Choline Kinase Beta*). *CPT1B* encodes an enzyme that is required for transport of long-chain fatty acids from the cytoplasm to the mitochondria while *CHKB* codes for an enzyme involved in phospholipid metabolism [30]. The main *CPT1B* TSS is only 0.6 kb from the 3′ end of *CHKB* (Figure 4). The 5′ end of *CPT1B* displayed SkM/heart-associated promoter chromatin and DNaseI hypersensitivity unlike the 5′ end of *CHKB* (Figure 4A,B). SkM or heart hypomethylated DMRs in the body of the gene were seen only in *CPT1B* (Figure 4C). In contrast to the preferential expression of *CPT1B* in striated muscle, *CHKB* is expressed at lower levels in SkM and heart than in most other tissues (Figure 4D). However, the *CHKB* RNA levels are still considerable in SkM and heart (TPM, 18 and 28), which is consistent with *CHKB* being linked to myopathies [30]. Inverse correlations between levels of *CPT1B* RNA and *CHKB* RNA were seen for many tissues in addition to SkM and heart (Figure 4D).

Myoblasts transcribe much more *CHKB* than *CPT1B* but embryonic stem cells (ESC) highly express exons from both genes despite only the *CHKB* 5′-region displaying promoter chromatin (Figure 4E). There is a read-through RNA transcript from *CHKB* through *CPT1B (CHKB-CPT1B*) that is predicted to be a noncoding RNA (Figure 4A) due to nonsense-mediated RNA decay [30]. RNA-seq data for ESC and the lack of promoter chromatin at the 5′ end of *CPT1B* but its presence at the 5′ end of *CHKB* in these cells indicate much formation of the *CPT1B-CHKB* read-through transcript in pluripotent embryonic cells (Figure 4A,E). Myoblasts apparently have little or no read-through transcription, possibly due to their weak enhancer chromatin (yellow segment) at the 5′ end of *CPT1B* favoring termination of the *CHKB* RNA (Figure 4A,E).

### 2.4. Highly Specific Expression in Striated Muscle of CKMT2, SLC25A4 and ACO2 Is Associated with a Super-Enhancer over the Gene Body

**Figure 5:**
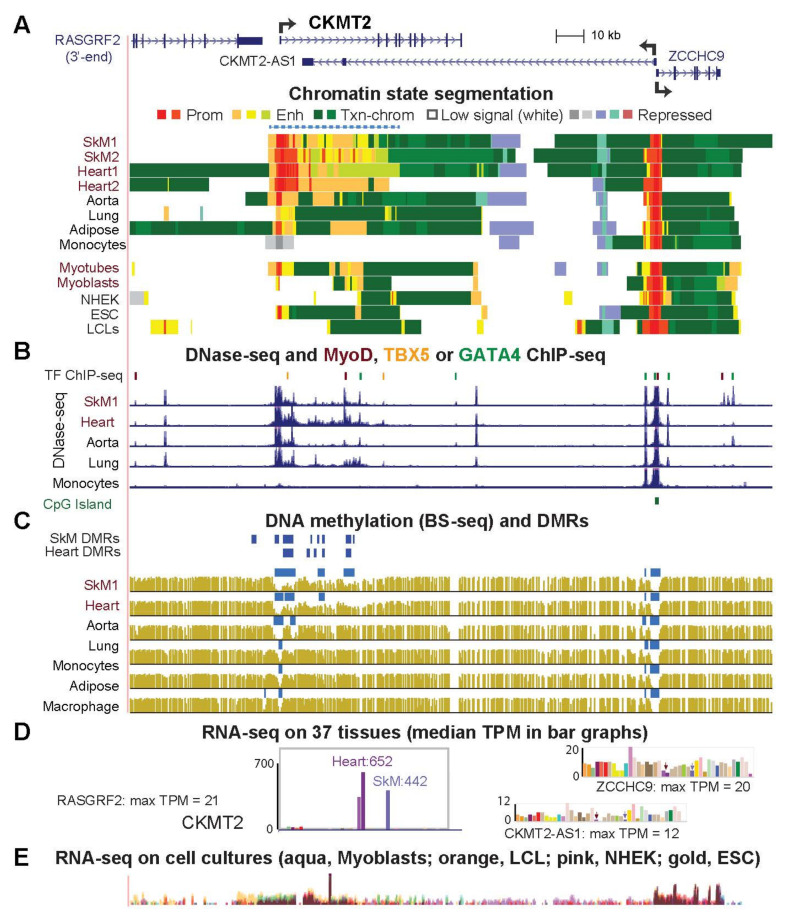
*CKMT2* exhibits a striated muscle-specific super-enhancer containing SkM and heart DMRs. *CKMT2*, *RASGRF2*, *ZCCH9* and the *CKMT2*-overlapping non-coding antisense transcript (chr5:80,502,017–80,618,428). (**A**–**E**), similar to Figure 3. Dotted line over the chromatin state tracks, super-enhancer in SkM and heart.

*CKMT2* (*Mitochondrial creatine kinase*), *SLC25A4/ANT* (*Solute Carrier Family 25 Member 4*), *ACO2* (*Aconitase 2*) encode mitochondrial proteins involved in transfer of phosphate from ATP, translocation of ATP from the mitochondrial matrix to the cytoplasm, or interconversions in the TCA cycle. They are highly expressed in SkM and heart (TPM > 250) and have higher expression than in 34 other tissue types (Table 1 and Table S1). Over much of the gene body, these three genes had extensive clusters of enhancer chromatin adjacent to promoter chromatin specifically in SkM and heart (Figure 5A, Figures S3 and S4, dotted blue line). Such clusters of strong enhancer chromatin (usually adjacent to promoter chromatin) are called super-enhancers and can be detected by tissue-specific enrichment in histone H3K27ac over a >5 kb region [40]. Super-enhancers are most often found at genes expressed at high levels in a strongly tissue-specific manner [40], as observed for these genes. DNA hypomethylation and DNaseI hypersensitivity associated with striated muscle and as well as MyoD binding in myoblasts and GATA4 and TBX5 binding in cardiomyocytes were seen at the super-enhancers (Figure 5A–C, Figures S3 and S4). *CKMT2, SLC25A4* and *ACO2* are expressed in myoblasts but only *CKMT2* shows preferential expression relative to other cell cultures (Table S3). Myotubes had ~200-fold more expression of *CKMT2* than myoblasts with parallel increases in promoter and enhancer chromatin (Figure 5; Table S4).

### 2.5. PRKN/PARK2, a Gene Important for Mitophagy, Has Striated Muscle-Associated and Brain-Associated Enhancer Chromatin and Encodes Myoblast/Skeletal-Related Antisense RNA at Its 3′ End

**Figure 6:**
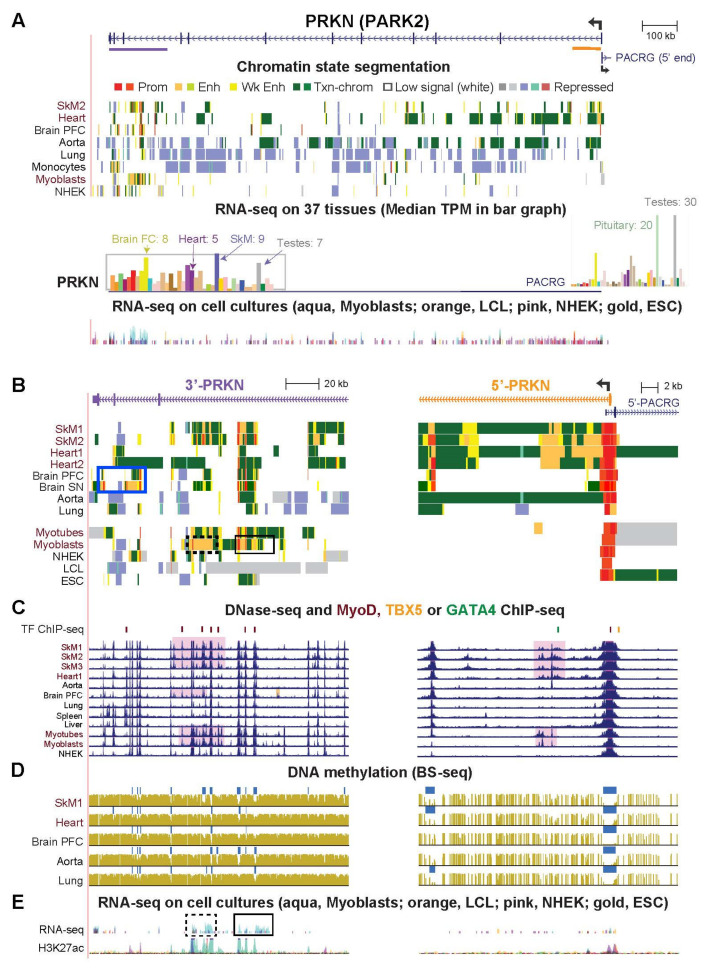
Epigenetic profile for *PRKN/PARK2* shows tissue-specific regulatory chromatin at the 5′- and 3′ ends of this 1.38-Mb gene. (**A**) The entire *PRKN* gene and part of the 5′ overlapping *PACRG* gene (chr6:161,720,965–163,176,926) with chromatin state and RNA-seq tracks. Purple or orange lines beneath the gene structure, regions amplified in the lower panels. (**B**–**E**) Left side, 3′ end (156 kb) of *PRKN* (chr6:161,765,976–161,922,054); right side, 5′ end (31 kb) (chr6:163,125,655–163,157,029). Pink highlighting in panel (**C**), DHS profiles specific to SkM, myoblasts, or brain. See Figure S5 for more details about boxed regions in panels (**B**,**E**). SN, substantia nigra; FC, frontal cortex.

*PRKN* (*PARK2, Parkin RBR E3 Ubiquitin Protein Ligase*; Figure 6) is an unusually large gene that codes for an E3 ubiquitin ligase (Parkin), which plays a major role in mitochondrial quality control as well as other regulatory roles [41]. Recessive loss-of-function mutations in this gene cause a juvenile form of Parkinson’s disease, which results in abnormal mitochondrial function in the substantia nigra, in skeletal muscle, and in platelets [10]. SkM and frontal cortex of brain were the tissues exhibiting the highest expression of this gene although their median expression levels were only TPM 9 and 8, respectively, and the gene is expressed in many other tissues (Figure 6A). Given the modest levels of expression of *PRKN* in SkM and heart (Table 1) and its 1.38-Mb size, it is not surprising that enhancer chromatin associated with the gene in these tissues looks sparse when viewing the whole gene in the UCSC Genome Browser [24]. Examination of subregions of the gene revealed distinctive enhancer chromatin, DHS, MyoD sites overlapping DHS, and DNA hypomethylation at the 3′ end of *PRKN* in SkM and myoblasts (Figure 6B, left and Figure S5) and in intron 1 in SkM and heart (Figure 6B, right). The epigenetic findings for myoblasts are consistent with their selective expression of this gene relative to heterologous cell cultures (Table S4).

We found that two regions of enhancer chromatin at the 3′ end of *PRKN* in myoblasts were associated with novel antisense (AS) intronic transcripts (Figure 6E, boxes, and Figure S5). Several polyadenylated AS transcripts from the distal end of the gene were indicated by the multiple promoter chromatin regions (Figure 6B, left), strand-specific RNA-seq profiles (Figure S5) and CAGE profiles (not shown). A myoblast-associated AS transcript aligned with the 15-kb myoblast-specific enhancer in this region (Figure 6B,E dotted boxes and Figure S5). In addition, other AS transcripts were seen preferentially in both SkM and myoblasts that align with the myoblast/SkM enhancer/promoter region near the end of the gene (Figure 6B,E, black box and Figure S5). Seven postnatal brain areas displayed enhancer and promoter chromatin similar to that of SkM. Importantly, there was also a region of brain-specific enhancer/promoter chromatin closer to the 3′ end of the gene that was seen in all seven brain regions (Figure 6B, left, blue box and Figure S5).

*PRKN* shares a bidirectional promoter with the 59-kb *PACRG* (*Parkin-coregulated gene*) (Figure 6A). Their TSS are only 0.2 kb apart. PACRG protein suppresses cell death caused by the accumulation of an unfolded protein substrate of Parkin [30]. Although the name of *PACRG* indicates coregulation with *PRKN*, we found only a few tissues that share similar expression profiles (notably brain and testis).

Functionally related to *PRKN* is another striated muscle and mitochondria-associated gene *VDAC1* (*Voltage-Dependent Anion Channel 1*; Figure S6). *VDAC1* encodes a major component of the outer mitochondrial membrane and is a substrate for poly-ubiquitination by Parkin. It is required for Parkin’s role in mitophagy in neural cells [42]. *VDAC1* displayed more intragenic DHS, enhancer chromatin (a super-enhancer), and DNA hypomethylation in SkM and heart than did heterologous tissues, findings that are consistent with striated muscle displaying the highest expression of this gene (Figure S6).

### 2.6. PPARGC1A, a Key Gene for Mitochondrial Biogenesis, Generates Multiple RNA Isoforms Whose Tissue Specificity Is Elucidated by Tissue-Specific Epigenetics and Novel Transcripts

**Figure 7:**
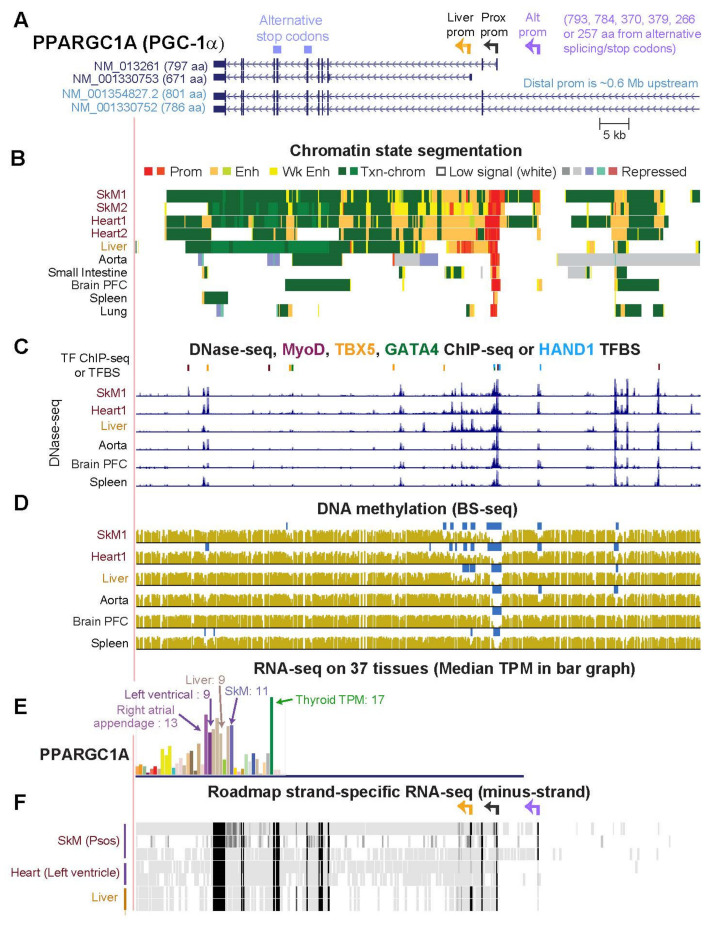
The tissue specificity of the epigenetic profiles at three of the four alternate promoter regions of *PPARGC1A/PGC-1α*. (**A**) Canonical isoform of *PPARGC1A* (NM_013261), a liver-associated isoform (NM_001330753), and part of two of the long isoforms are shown (chr4:23,766,778–23,961,817). The previously assigned names of the four alternative promoters for this gene are given. (**B**–**E**) Panels are as in Figure 2 and Figure 4. The liver-associated, proximal (Prox), and alternate (Alt) TSS are indicated by color-coded broken arrows. Although no CpG island was identified, there was a high CpG content at the Prox TSS for this gene. (**F**) RNA-seq for the minus-strand on tissues from the Roadmap Epigenomics Project for a 34-yo male, 30-yo female, and 3-yo male psoas; left and right ventricles of a 3-yo male, and technical duplicate liver samples from a 3-yo male. See Figure S7 for the full-length gene.

*PPARGC1A* (*PGC-1α, PPARG Coactivator 1 Alpha*), is another striated muscle/mitochondria-associated gene involved in Parkinson’s disease [43]. PGC-1α is a transcriptional coactivator that controls transcription initiation, pre-mRNA elongation, and RNA splicing to regulate mitochondrial biogenesis, homeostatic glucose utilization in striated muscle and liver, brown adipocyte differentiation, and various other pathways [44,45]. Much of the versatility of this co-regulator in its tissue-specific roles can be ascribed to its multiple tissue-specific mRNA isoforms and corresponding protein isoforms. PGC-1α protein isoforms differ in biochemical properties, protein-protein interactions, stability, and subcellular localization [46,47,48,49]. Isoform formation depends partly on the choice of one of the following promoters for transcription initiation: the proximal (Prox) promoter (encoding the canonical 797-aa PGC-1α1), the 14-kb upstream alternate (Alt) promoter, the downstream liver-associated promoter, and a ~0.6-Mb upstream brain-associated promoter (distal promoter) (Figure 7A and Figure S7) [43,46]. Despite documentation of the SkM-associated Alt promoter [46,50], the corresponding TSS is not indicated by any RefSeq (Figure S7A) or Ensembl (V38) gene structure. Usage of the Alt promoter affects not only the N-terminal polypeptide sequence, but also determines alternative splicing and stop codons to generate diverse polypeptides, e.g., PGC-1α2 (379 aa), PGC-1α3 (370 aa) or PGC-1α4 (266 aa). Promoter chromatin was observed at the Alt TSS specifically in the psoas sample (SkM1) while enhancer chromatin at the same location was seen in the leg-muscle sample (SkM2, Figure 7B). A DHS and DNA hypomethylation were observed at the Alt TSS in SkM (Figure 7C,D). The lack of MyoD binding to the Alt prom in myoblasts (Figure 7C) may be attributable to their very low level of expression of this gene (Table S4) in contrast to its higher expression in SkM and heart than in most other tissues.

**Figure 8:**
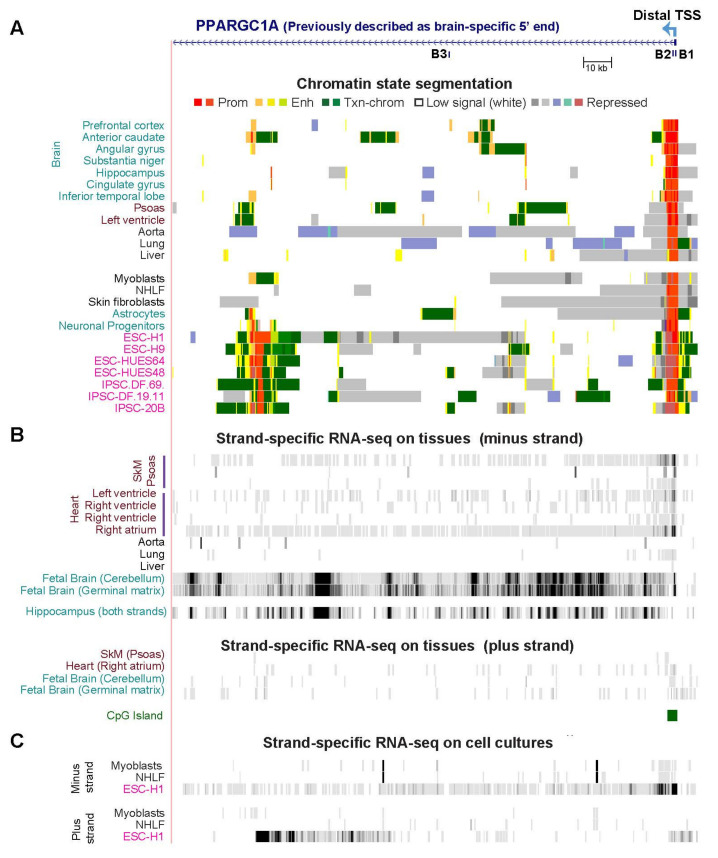
The far-distal end of *PPARGC1A* associated with brain isoforms contains novel antisense promoter chromatin in embryonic stem cells (ESC) and induced pluripotent stem cells (iPSC) and novel exon-like signal from the sense strand in brain samples. (**A**) Chromatin state segmentation tracks in the 178-kb far-distal 5′-region of *PPARGC1A* (chr4:24,295,195–24,482,165; from 7 kb upstream of the distal TSS) that contains the brain-associated distal promoter and non-coding exons B1, B2, and B3 [53]. The distal TSS is ~0.6 Mb upstream of the Prox, Alt, and Liver TSS in Figure 7. Profiles from four ESC cell lines and three iPSC derived from fibroblasts are shown. (**B**) Roadmap Epigenome Project-derived strand-specific RNA-seq using poly(A)^+^ RNA as in Figure 7. (**C**) ENCODE Project-derived RNA-seq using poly(A)^+^ RNA including the prominent ESC AS RNA.

Strand-specific RNA-seq indicated more usage of the Alt prom in three psoas muscle samples than in other tissues (Figure 7F and data not shown) consistent with the previous association of this promoter with SkM, especially in certain physiological states [49,51,52]. Transcription from the Alt promoter can give protein isoforms that upregulate mitochondrial biogenesis or ones that only upregulate other pathways. Two psoas samples (34-yo male and 30-yo female) used the Alt and Liver promoters as well as the Prox promoter and the third (3-yo male) used only the Prox TSS and Alt promoters, as indicated by their RNA-seq profiles (Figure 7F). Near the four promoter regions of *PPARGC1A* were enhancer chromatin segments, DHS, DNA hypomethylation, and myoblast or cardiomyocyte TF binding sites that were consistent with the SkM-, heart-, liver-, or brain-associated usage of these promoters (Figure 7, Figure 8A and Figure S8).

Surprisingly, strand-specific RNA-seq suggests that the distal TSS, which is ~0.6 Mb upstream of the three other TSS and has been associated with brain [43], initiates some of the *PPARGC1A* transcripts in certain SkM and heart samples as well as being the major promoter for *PPARGC1A* in ESC (Figure 8, Figures S7 and S8). Possibly related to this ESC finding, ESC displayed a novel lncRNA specifically from the AS strand (plus-strand) 148 kb downstream of the distal TSS and immediately downstream of ESC-specific AS promoter chromatin and an ESC-specific DHS (Figure 8A,C and Figure S7). Moreover, this promoter chromatin region which apparently drives expression of the intragenic ESC AS transcript was also seen in all examined induced pluripotent stem cell (iPSC) lines (Figure 8A; iPSC RNA-seq, not available). It was not observed in 17 other cell cultures including ESC-derived mesenchymal stem cells and ESC-derived endodermal cells but was in HeLa cells, ESC-derived ectodermal cells, and, to a small extent, in astrocytes, neuronal progenitor cells, and brain anterior caudate (Figure 8A and data not shown).

Approximately 29 kb upstream of the *PPARGC1A* distal TSS, Soyal et al. [53] noted a spliced expressed sequence tag (EST; CX755957) from a human blastocyst-derived pluripotent stem cell line (upstream of the region shown in Figure 8). In the four ESC and two iPSC cultures, but not in other cell or tissue types, we found promoter chromatin upstream of CX755957 near enhancer chromatin (data not shown). In the one ESC with available strand-specific RNA-seq profiles, there was a plus-strand transcript in the same region, although not with the same exonic structure, as for CX755957 (not shown). Soyal et al. also described a minus-strand ESC-derived spliced EST (CN283176) encoded by a 1.3-kb sequence and overlapping the first exon (noncoding exon B1 [53]) at the distal TSS. As seen in Figure 8 and Figure S8, there was poly(A)^+^ RNA-seq signal at exon B1 in ESC but it was broader than exon B1 and extended further downstream. This additional minus-strand RNA signal immediately downstream of the B1/B2 exons in ESC and also in some striated muscle samples is of uncertain significance.

We also examined the far-distal region of *PPARGC1A* for brain-specific isoforms [53]. From fetal (20-week) cerebellum and germinal matrix samples, the two brain tissues for which strand-specific RNA-seq was available, minus-strand RNA-seq revealed many novel exon-like signals in addition to the previously reported exon B1, B4, and B5 signals [53] in the 0.5-Mb region downstream of the distal promoter (Figure 8B and Figure S7). In contrast, negligible signal in this region was seen from plus-strand-specific RNA-seq profiles (Figure 8B). None of the other >40 examined tissue or cell samples exhibited these novel sense-strand exon-like RNA signals except for a neurosphere sample (15 wk embryo originated from ganglionic eminence; data not shown). The novel RNA signals showed little overlap with enhancer or promoter chromatin in brain (Figure 8A). One additional poly(A)^+^ RNA-seq brain profile was available, namely for hippocampus from an 80-yo, but this RNA analysis was done without strand separation. This sample displayed many exon-like transcribed regions similar to those of the two fetal samples in the 0.3-Mb region downstream of the distal TSS (Figure 8B and Figure S7). Therefore, the novel sense exon-like RNA signals are found in postnatal brain as well as fetal brain.

## 3. Discussion

The overrepresentation of mitochondrial GO associations for genes preferentially expressed in both SkM and heart but not aorta (Figure 1) is consistent with the special importance of mitochondria to striated muscle [11,21,24,25,29,54] and the frequent positive or negative disease-associations of these genes (Table S5; [55,56,57,58,59,60]). Many of the 27 striated muscle/mitochondria-associated genes displayed SkM/heart-associated enhancer chromatin that was intragenic, promoter-upstream, intergenic and downstream, or a super-enhancer spanning the gene (Figure 2, Figure 3, Figure 4, Figure 5, Figure 6 and Figure 7 and Figures S1–S8). Moreover, there were strong associations of the tissue-specific enhancer and promoter chromatin profiles with tissue-specific expression profiles. This epigenetic/transcription correlation indicates that transcription control plays a major role in determining the steady-state levels of mRNA for the studied genes. If this were not the case and tissue-specific posttranscriptional control had been superimposed on broad transcription profiles, we would not have seen the many examples of tissue-specific promoter/enhancer/open chromatin/DNA methylation profiles corresponding to expression profiles. Reports of epigenetic changes correlated with disease or altered physiological states usually focus on changes at promoters, which are easier to locate on the genome (e.g., [24,61,62,63,64]), although there are noteworthy exceptions (e.g., [65]). Our study reinforces the importance of testing for disease- and physiology-linked changes in chromatin and DNA epigenetics also at enhancers, which can show more extensive tissue-specific differences correlated with expression than do promoters (Figure 3, Figure 6, Figures S3, S4 and S6).

We studied not only the epigenomics and transcriptomics of the SkM/heart/mitochondria-associated genes but also of their neighboring genes. Important intergenic epigenetics/transcription relationships were thereby discovered. For the striated muscle-specific *COX6A2*, SkM/heart-associated enhancer chromatin was found at two adjacent genes (*ITGAD* and *ZNF843*) that are not expressed preferentially in SkM or heart (Figure 2). This suggests that intragenic enhancer chromatin from *ITGAD* and *ZNF843* upregulated the adjacent *COX6A2* rather than *ITGAD* and *ZNF843* [23]. The gene neighbors (5′-to-3′) *CPT1B* and *CHKB* displayed an inverse correlation between their expression in most tissues, including SkM and heart, unlike a previous report about five mouse tissues [66]. Myoblasts express mostly just *CHKB* (which fosters formation of the inner mitochondrial membrane) while ESC cells predominantly express the read-through *CHKB-CPT1B* transcript initiated at the *CHKB* promoter (Figure 4). In contrast, SkM and heart express much more *CPT1B* (essential for fatty acid oxidation and contributing to ATP synthesis) but still have considerable levels of *CHKB* RNA (Figure 4D,E). We propose that a highly active *CPT1B* promoter in striated muscle inhibits read-through transcription from the upstream *CHKB* gene and downmodulates *CHKB* promoter activity but still allows production of enough mRNA from both genes for their critical roles in skeletal muscle and heart [30,66].

Among the 27 striated muscle/mitochondria-associated genes (Table 1), four (*COX6A2*, *COX5A*, *COX10* and *COX7A1*) encode subunits of cytochrome c oxidase, the terminal enzyme of the electron transport chain [67]. Mutations of these four genes are associated with various congenital syndromes that include SkM and/or cardiac abnormalities [30] (Table S5). *COX6A2* and *COX7A1* have paralogs on different chromosomes, *COX6A1* and *COX7A2,* respectively. *COX6A1* and *COX7A2* encode similar polypeptides to those of *COX6A2* and *COX7A1*, but they have a much broader tissue expression profile and lack specificity for striated muscle [28,30,68]. The striated muscle specificity and level of expression of *COX6A2* is exceptionally high (Table 1) in contrast to its paralog *COX6A1*, which has its lowest levels of expression among adult tissues in SkM and heart [28]. This illustrates the need for muscle-specific paralogs of some genes with highly muscle-specific roles. For the striated muscle-specific *COX7A1* (Figure S1), advanced age is significantly associated with lower expression and increased methylation in exon 1 at a CpG island [64]. This finding may reflect genome-wide increases in CpG island methylation with age observed in human leukocytes [69]. Steady-state levels of *COX7A1* mRNA in SkM are significantly associated with glucose uptake and total-body aerobic capacity [64]. These reports and the correlation between tissue-specific profiles of gene expression and DNA hypomethylation at and adjacent to the *COX7A1* CpG island (Figure S1) suggest that DNA methylation changes in response to aging and physiological alterations help regulate or stabilize transcription levels at this gene.

Some of the genes that we studied which are preferentially expressed in striated muscle are not predominantly localized to the mitochondria. Prominent among these are *PPARGC1A,* which encodes transcription coregulator PGC-1α, whose main function is enabling mitochondrial biogenesis and optimal mitochondrial function, and *PRKN/PARK2*, which codes for Parkin, a ubiquitin ligase whose main function is to upregulate a major pathway for mitophagy [42,46]. Mitophagy is critical to maintaining proper SkM function and to restorative processes after cardiac or SkM stress or injury and in response to sarcopenia [17,70,71]. There are also biochemical *PRKN-PPARGC1A* connections, e.g., Parkin polyubiquitinates ZNF746, a repressor of *PPARGC1A* expression, which marks the repressor for degradation and, thereby, promotes transcription of *PPARGC1A* [72]. Mutations in *PRKN* cause a form of autosomal recessive Parkinson’s disease, which is characterized by cytoplasmic inclusions in the brain. These inclusions contain aggregation-prone proteins and sometimes organelles, including mitochondria [42,73]. Although the primary target of Parkin-associated Parkinson’s disease is the brain, this enzyme also has special importance for SkM and cardiac muscle and, like PGC-1α, has regulatory roles that are not restricted to mitochondria [41,46,47,74].

The prominent and varied functions of Parkin and PGC-1α are reflected in the intricate and tissue-specific transcriptomics and, in our study, in the epigenomics of their genes. At the 1380-kb *PRKN* gene, chromatin state, DNaseI hypersensitivity, MyoD binding, DNA methylation profiles, and transcription profiles suggest that that the little-studied [65] ~100 kb region in the 3′ end of the gene plays a more important role in transcription control than does the central gene region. We propose that a novel myoblast-specific AS RNA from the distal myoblast-specific 15 kb enhancer chromatin region in intron 9 of *PRKN* (Figure 6 and Figure S5) is involved in upregulating the *PRKN* promoter ~1.3 Mb upstream to fill a requirement for large amounts of Parkin in the early stages of myoblast differentiation to myotubes. Myoblasts need to prepare for a transition from a highly glycolytic state to a state where they rely mainly on oxidative phosphorylation (OXPHOS) for their increased energy needs in myotubes. This change in energy needs is accompanied by mitochondrial clearance via mitophagy followed by mitochondrial biogenesis to provide mitochondria that are more active in OXPHOS during myoblast differentiation [75]. Although cardiomyocyte differentiation from precursor cells was found to use a mitophagy pathway not involving Parkin and those precursor cells contained no detectable *PRKN* RNA before or after differentiation [76], we found preferential expression of *PRKN* in myoblasts vs. heterologous cell types. Moreover, the Parkin pathway has been implicated in mitophagy in myoblasts as well as in SkM [77]. It is likely that the mixed enhancer and promoter chromatin region that we found in myoblasts near the 3′ end of *PRKN* and the myoblast-specific AS transcription in this region upregulate *PRKN* transcription, as generally observed for some other enhancer regions that generate unidirectional poly(A)^+^ lncRNAs [78].

Additional enhancer/promoter regions in the 3′ end of the *PRKN* gene displayed tissue specificity, most notably for brain. Enhancer chromatin in various subregions in the last three introns of *PRKN* as well as the SkM/heart-specific enhancer chromatin immediately downstream of the TSS (Figure 6 and Figure S5) might be critical not only for tissue/cell type-specific differences in expression of this gene, but also for its responses to alterations in cell or tissue physiology. These findings are of clinical interest in view of evidence for *PRKN* dysregulation contributing to idiopathic Parkinson’s disease and other neurological diseases [79].

PPARGC1A/PGC-1α, as a master regulator of energy metabolism [45], has regulatory interactions with products of some of the SkM/heart/mitochondria genes that we studied, e.g., *PKRN*, *COQ10A* and *CPT1B* [20,80,81]. The PGC-1α protein isoforms derive partly from the alternative use of four different promoters in a 0.6 Mb region, which influences splicing patterns and stop-codon usage (Figure 7 and Figure S7). Specific isoforms of PGC-1α can differentially change both levels of transcription and types of splicing of target genes [48,52]. PGC-1α is a cofactor for various TFs and regulates mitochondrial biogenesis and respiration, fatty acid oxidation [80], and muscle fiber-type composition [82]. It also controls angiogenesis, muscle hypertrophy through downstream effects on myostatin levels (PGC-1α4 isoform from the Alt promoter), inhibition of apoptosis (PGC-1α4), innate immunity (PGC-1α4), gluconeogenesis (PGC-1α1 isoform from the Prox promoter), fasted-liver associated transcription (Liver promoter), and brain-associated gene expression (especially from the distal promoter; Figure 7A) [46,47,48]. Physiological effects on *PPARGC1A* transcription and epigenetics are illustrated by the finding that one round of strenuous exercise in the mouse was found to increase transcription from the Alt promoter but not the Prox promoter and to increase H3K4me3 methylation significantly in a 2-kb region centered around the Alt TSS [63].

We found RNA-seq evidence for the unexpected use of the Liver promoter and the 0.6-Mb upstream brain-associated distal promoter of *PPARGC1A* in several SkM samples. This is consistent with epigenetic marks and SkM- or heart-associated TFs binding in their vicinity in progenitor muscle cells (Figure 7B,C, Figures S7 and S8). In addition, in embryonic cells, we observed an ESC-associated (and iPSC-associated) AS promoter and a corresponding ESC-specific AS transcript (Figure 8) 148 kb downstream of the distal TSS and 0.43 Mb upstream of the other three *PPARGC1A* TSS. The epigenomic and transcriptomic profiles in ESC at this gene (Figure 8C, Figures S7 and S8) suggest that the intragenic AS transcription helps drive the predominant use of the distal promoter in ESC. We further propose that there are special functions for PGC-1α encoded by *PPARGC1A* mRNA initiated at the distal promoter in some non-neural cell types, including in early embryonic cells, in addition to the documented brain-specific functions [48,53]. Because some promoter chromatin was seen in the region of the intragenic ESC AS promoter in one brain sample (accompanied by enhancer and transcription-type chromatin), in astrocytes, and in neuronal progenitor cells, the intragenic AS promoter chromatin might also help regulate distal promoter usage in some brain cells.

Novel brain-specific sense-strand poly(A)^+^ transcripts from *PPARGC1A* were also observed in this study. They were seen at many exon-like regions scattered in a ~0.5 Mb region downstream of the distal promoter (Figure 8 and Figure S7) in addition to the previously described brain-associated *PPARGC1A* exons B1–B5 in this region [53]. We propose that these novel discrete RNA signals represent brain-specific lncRNAs that may originate at brain-associated exon B1 but may not extend into the canonical part of the *PPARGC1A* gene and so were not previously detected. These findings, the unexpected use of the distal and Liver promoters supplementing the Prox and Alt promoters of *PPARGC1A* in SkM, and the preferential use of the distal promoter in ESC need follow-up investigation given the importance of *PPARGC1A* transcription levels and isoform usage to exercise, aging, SkM hypertrophy, and disease, including facioscapulohumeral muscular dystrophy, Alzheimer’s disease, amyotrophic lateral sclerosis, Huntington’s disease, and ischemia [50,51,63,83,84,85,86].

## 4. Materials and Methods

### 4.1. RNA-Seq for Tissues and Cells

RNA levels (TPM, transcripts per million) for human tissues were from the GTEx RNA-seq database V8 [28], which contains 54 tissue types. Because tissues from brain are overrepresented in the dataset, we excluded 11 brain tissues other than prefrontal cortex (PFC) and cerebellum. We also excluded TPM values for two cell cultures and several partly redundant tissue types. For genes with more than one isoform, except where otherwise noted, only the main transcribed isoform is shown in the figures, as deduced from GTEx isoform expression data. The quantitation of RNA-seq data (FPKM, fragments per kilobase million) for human cell cultures was previously described for ENCODE data and for our own data comparing RNA levels in myoblasts and myotubes [26,87]. RNA-seq tracks, like all the tracks in gene figures, were visualized in the UCSC Genome Browser in hg19 [32] with lift-over from hg38 for tracks available only there (tissue RNA-seq and UniBind).

### 4.2. Epigenomics

The 18-state Roadmap Epigenomics [27] chromatin state segmentation analysis (chromHMM and AuxilliaryHMM) was used for determination of chromatin states. Strong enhancer or promoter chromatin displays a moderate or strong signal for both H3K27ac and H3K4me1 or for both H3K27ac and H3K4me3, respectively. Enhancer chromatin refers to States 6–10 (orange or yellow-green) and weak enhancer chromatin to State 11 (yellow) while promoter chromatin (red) is States 1, 2 or 4 and mixed promoter and enhancer chromatin (red) is State 3 [27]. Bisulfite-seq profiles of genome-wide CpG methylation and the DNaseI-hypersensitivity profiles were also from the RoadMap Epigenomics Project [27]. The tissue sources for chromatin and DNA epigenetics were previously described [27,88]. SkM1 refers to psoas (combined 3 yo and 34 yo) and SkM2 and SkM3 to upper leg muscle from a 72-yo female and a 54-yo male, respectively. Super-enhancers were assessed by dbSUPER [40] or SEdb (SEdb-a comprehensive human Super-Enhancer database (licpathway.net, July 2021) and confirmed by looking at the H3K27ac track in the UCSC Genome Browser [32]. Low methylated regions (LMRs) shown in the figures refer to regions with significantly lower DNA methylation than in the rest of the same genome as determined by Song et al. [33]. We determined SkM and heart DMRs by comparison of bisulfite-seq (whole-genome bisulfite sequencing) data for SkM or heart vs. aorta, lung, monocytes, and adipose tissue as previously described [22].

### 4.3. Transcription Factor Binding

MyoD, GATA4, and TBX5 binding sites in myoblasts were determined from UniBind data tracks (Version 2021, robust) in the UCSC browser for hg38 [32]. The cardiomyocytes used for GATA4 and TBX5 ChIP-seq in the UniBind database were induced pluripotent stem cell (iPSC)-derived cardiomyocytes that were from cultures capable of spontaneous contraction and were purified to give >90% cardiomyocytes as deduced from immunostaining for cardiac-specific troponin T2 [89]. Because of the lack of NKX2-5 and HAND1 ChIP-seq profiles for heart-related samples, we used a TF-binding prediction program (Transfac https://portal.genexplain.com/ (Version 2021.2; vertebrate sites, all matrices, and minimize false positives) for predicted binding sites within DNaseI hypersensitive sites in left ventricle. The position weight matrix (PWM) for HAND1 binding sites was for HAND1::E47; MYOD1 also can heterodimerize with E47 to give MyoD TF [35].

## 5. Conclusions

Genes associated with mitochondrial function, structure, biogenesis, or mitophagy are overrepresented among the category of genes preferentially expressed in both skeletal muscle and heart. Many of these genes are of great importance to metabolism, health, aging and physiological changes. The tissue-specific epigenetic profiles of these genes often paralleled their expression profiles and indicated some unexpected locations of positive regulatory elements, such as, in the gene body 1.28 Mb downstream of the 5′ end of 1.38-Mb *PRKN*/*PARK2* gene or within the gene neighbor of a studied gene (e.g., *COX6A2* and *ITGAD* or *ZNF843*). In addition, novel intragenic antisense lncRNAs were found within *PRKN* in myoblasts and skeletal muscle and within *PPARGC1A/PGC-1α* in embryonic stem cells. This study exemplifies the need to look beyond canonical promoters to understand the regulation of expression of genes, in general, and these 27 striated muscle-associated genes, in particular.

## Figures and Tables

**Table 1:** Genes that are preferentially expressed in both skeletal and heart and are associated with mitochondria ^a^.

Gene	TPM ^b^ SkM	TPM Heart	TPM Ratios: SkM/Heart	TPM Ratios: SkM/Aorta	Median TPM of Other Tissues	TPM Ratios: SkM/Median Other Tissues	TPM Ratios: Heart/Median Other Tissues
ACADS ^c^	112.8	60.1	1.9	5.2	30.7	3.7	2.0
ACO2 (Fig. S4)	272.4	252.4	1.1	5.4	53.8	5.1	4.7
CHCHD10 ^c^	229.7	248.5	0.9	7.5	45.7	5.0	5.4
CKMT2 (Fig. 5)	441.9	652.4	0.7	17.7	5.6	79.5	117.4
COQ10A (Fig. 3)	65.1	79.1	0.8	5.0	14.0	4.6	5.6
COX5A ^c^	389.6	421.1	0.9	5.4	88.3	4.4	4.8
COX6A2 (Fig. 2)	2283.9	1593.7	1.4	556.2	1.0	2263.4	1579.4
COX7A1 (Fig. S1)	704.8	584.3	1.2	4.3	56.5	12.5	10.3
COX10 ^c^	28.6	19.1	1.5	3.8	9.5	3.0	2.0
CPT1B (Fig. 4)	100.5	144.6	0.7	13.8	20.9	4.8	6.9
G0S2 ^c^	65.0	84.9	0.8	3.6	22.8	2.8	3.7
GOT1 ^c^	212.3	304.6	0.7	10.5	32.7	6.5	9.3
GOT2 ^c^	280.0	251.9	1.1	4.7	74.9	3.7	3.4
HADHB (Fig. S2)	375.3	307.5	1.2	5.6	77.1	4.9	4.0
IDH2 ^c^	442.8	311.5	1.4	13.5	54.1	8.2	5.8
LDHD ^c^	47.1	58.5	0.8	4.5	12.0	3.9	4.9
MDH2 ^c^	361.2	210.3	1.7	3.4	104.6	3.5	2.0
NDUFB9 ^c^	299.5	188.2	1.6	3.2	82.8	3.6	2.3
NDUFS1 ^c^	50.6	42.4	1.2	3.1	16.3	3.1	2.6
NNT ^c^	69.7	52.5	1.3	4.2	16.4	4.3	3.2
OGDH ^c^	222.2	201.4	1.1	3.1	65.6	3.4	3.1
PPARGC1A (Figs. 7, 8, S7 and S8)	11.0	9.4	1.2	24.5	2.7	4.2	3.5
PRKN/PARK2 (Figs. 6 and S5)	8.7	4.8	1.8	3.4	2.4	3.6	2.0
SLC25A4 (Fig. S3)	370.5	563.8	0.7	3.4	24.6	15.0	22.9
TMEM143 ^c^	34.2	21.1	1.6	4.9	8.5	4.0	2.5
UQCRFS1 ^c^	133.8	102.0	1.3	4.0	39.5	3.4	2.6
VDAC1 (Fig. S6)	390.9	199.3	2.0	3.7	91.0	4.3	2.2

^a^ Preferential expression defined by flow scheme (Figure 1); ^b^ TPM, transcripts per kilobase millions; SkM, skeletal muscle; other tissues, 34 tissues other than SkM or heart; Fig., Figure; ^c^ not very tissue-specific epigenetics.

## Data Availability

Data are available at the UCSC Genome Browser (http://www.genome.ucsc.edu/ including the RoadMap Epigenomics Analysis Integrative Hub and the DNA Methylation Analysis Hub) and at the GTEx Portal.

## Supplementary Materials

The following are available online at https://www.mdpi.com/article/10.3390/epigenomes6010001/s1, Figure S1: Striated muscle-specific DNA hypomethylation extending over the gene body of COX7A1 and downstream is associated with the gene’s preferential expression in striated muscle. Figure S2: HADHB and its co-expressed gene neighbor, HADHA, share a bidirectional promoter with highest expression and DNA hypomethylation in skeletal muscle. Figure S3: Preferential expression of SLC25A4/ANT1 is correlated with an overlapping super-enhancer containing DNA hypomethylated subregions in skeletal muscle and heart. Figure S4: Preferential expression of ACO2 is correlated with an overlapping a super-enhancer containing DNA hypomethylated subregions in skeletal muscle and heart. Figure S5: The myoblast-specific lncRNA from the 3′ end of PRKN is an antisense transcript. Figure S6: Preferential expression of VDAC1 is correlated with an overlapping super-enhancer containing DNA hypomethylated subregions in skeletal muscle and heart. Figure S7: The full-length PPARGC1A gene including the far distal promoter displays novel brain and embryonal stem cell RNA signals. Figure S8: Closer view of the promoter regions of PPARGC1A shows tissue-specific epigenetic signatures. Table S1: Genes preferentially expressed in skeletal muscle (SkM) and heart but not in aorta. Table S2: DAVID analysis of the 97 genes shown in Table S1. Table S3: Expression of gene neighbors of highlighted striated muscle/mitochondria-associated genes. Table S4: Expression of highlighted striated muscle/mitochondria-associated genes. Table S5: Function and disease association of highlighted striated muscle/mitochondria-associated genes.
