# Supplementary material for: Epigenetics of Mitochondria-Associated Genes in Striated Muscle
Source: Epigenomes. 2021 Dec 22;6(1):1. doi: 10.3390/epigenomes6010001 (PMC8788487; doi:10.3390/epigenomes6010001)
Supplement: Supplementary file 1 [file epigenomes-06-00001-s001.zip › epigenomes-1484731-supplementary/EhrlichK_Epigen_StriatedMuscle_Mitoch_Genes_Suppl_Figs__12_3_21.pdf]

Epigenetics of mitochondria-associated genes in striated muscle

Kenneth C. Ehrlich, Hong-Wen Deng, and Melanie Ehrlich

Supplementary Figures

Figure S1 Striated muscle-specific DNA hypomethylation extending over the gene body of *COX7A1* and downstream is associated with the gene's preferential expression in striated muscle.

Figure S2. *HADHB* and its co-expressed gene neighbor, *HADHA*, share a bidirectional promoter with highest expression and DNA hypomethylation in skeletal muscle.

Figure S3. Preferential expression of *SLC25A4/ANT1* is correlated with an overlapping super-enhancer containing DNA hypomethylated subregions in skeletal muscle and heart.

Figure S4. Preferential expression of *ACO2* is correlated with an overlapping a super-enhancer containing DNA hypomethylated subregions in skeletal muscle and heart.

Figure S5. The myoblast-specific lncRNA from the 3′ end of *PRKN* is an antisense transcript.

Figure S6. Preferential expression of *VDAC1* is correlated with an overlapping super-enhancer containing DNA hypomethylated subregions in skeletal muscle and heart.

Figure S7. The full-length *PPARGC1A* gene including the far distal promoter displays novel brain and embryonal stem cell RNA signals.

Figure S8. Closer view of the promoter regions of *PPARGC1A* shows tissue-specific epigenetic signatures.

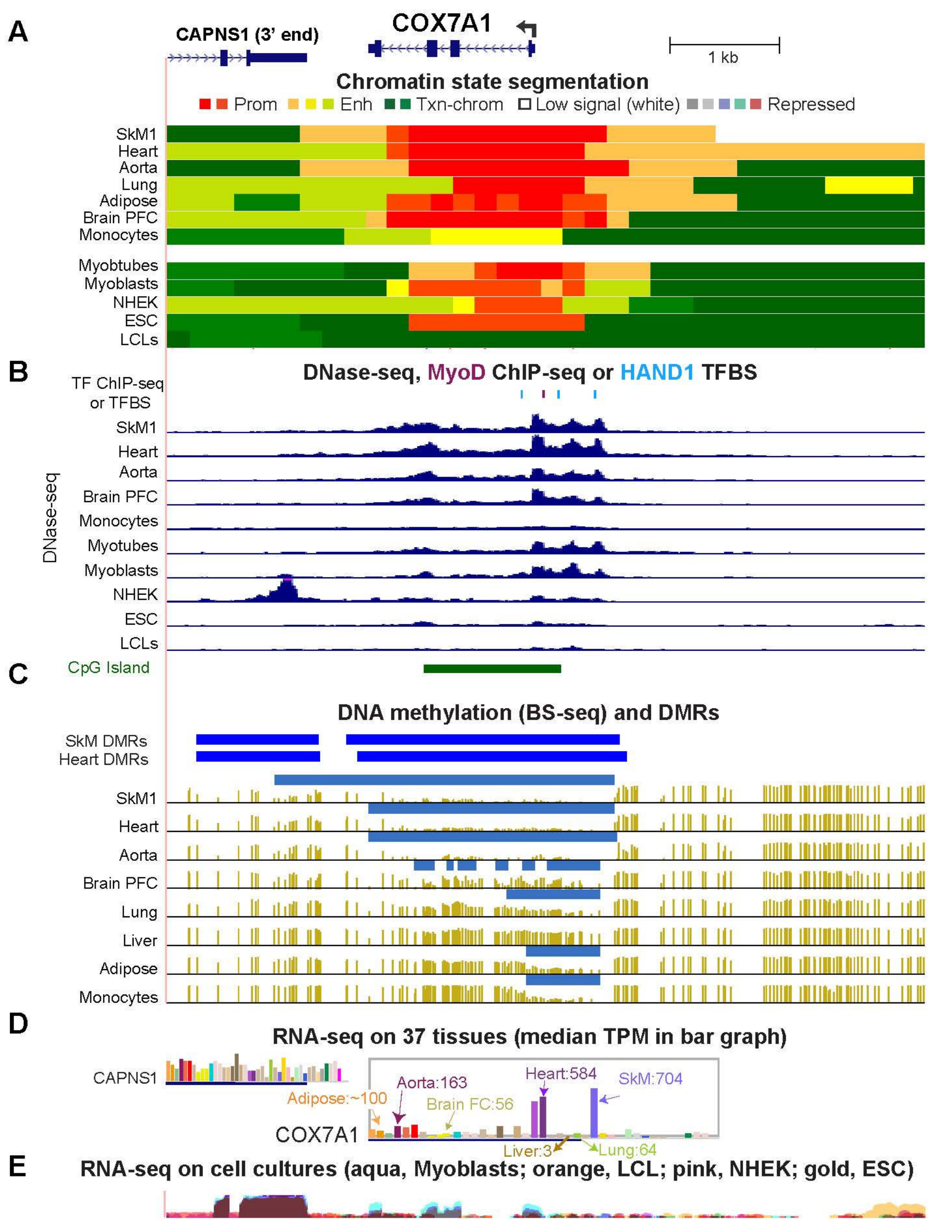

Figure S1 Striated muscle-specific DNA hypomethylation extending over the gene body of *COX7A1* and downstream is associated with the gene's preferential expression in striated muscle. (A) *COX7A1* gene neighborhood (chr19:36,639,976-36,646,905, hg19). Color-coded chromatin state segmentation (Roadmap Epigenomics Project; 18-state). **(B)** DNase-seq and MyoD binding as determined from MyoD ChIP-seq (UniBind) and HAND1 TFBS (Transfac prediction program, https://genexplain.com/transfac/); only TF sites at DNaseI hypersensitive sites (DHS) associated with skeletal muscle are shown. **(C)** Whole genome bisulfite-seq for DNA methylation levels; a CpG island in this region indicated by the green box. Significant SkM and heart hypomethylated DMRs were determined by comparison of these tissues to aorta, lung, adipose tissue, and monocytes. Blue bars, regions of low-methylation (LMRs) relative to methylation throughout the genome in the same tissue. **(D)** The GTEx RNA-seq expression profile is shown as bar graphs with linearly displayed median values for TPM from hundreds of biological replicates for each tissue type unless otherwise indicated (https://gtexportal.org/home/). **(E)** RNA-seq on cell culture-derived poly(A)$^{+}$ RNA is shown as an overlay of the indicated five cell types (log scale). All tracks are horizontally aligned and in hg19 coordinates. TPM, transcripts per kilobase millions; Prom, promoter; Enh, enhancer; Txn, transcription; SkM1, psoas skeletal muscle; SkM2, skeletal muscle from leg; PFC, prefrontal cortexDMR, differentially methylated region; NHEK, normal human epithelial kidney cells; ESC, embryonic stem cells (H1); LCL, lymphoblastoid cell line; FC, frontal cortex.

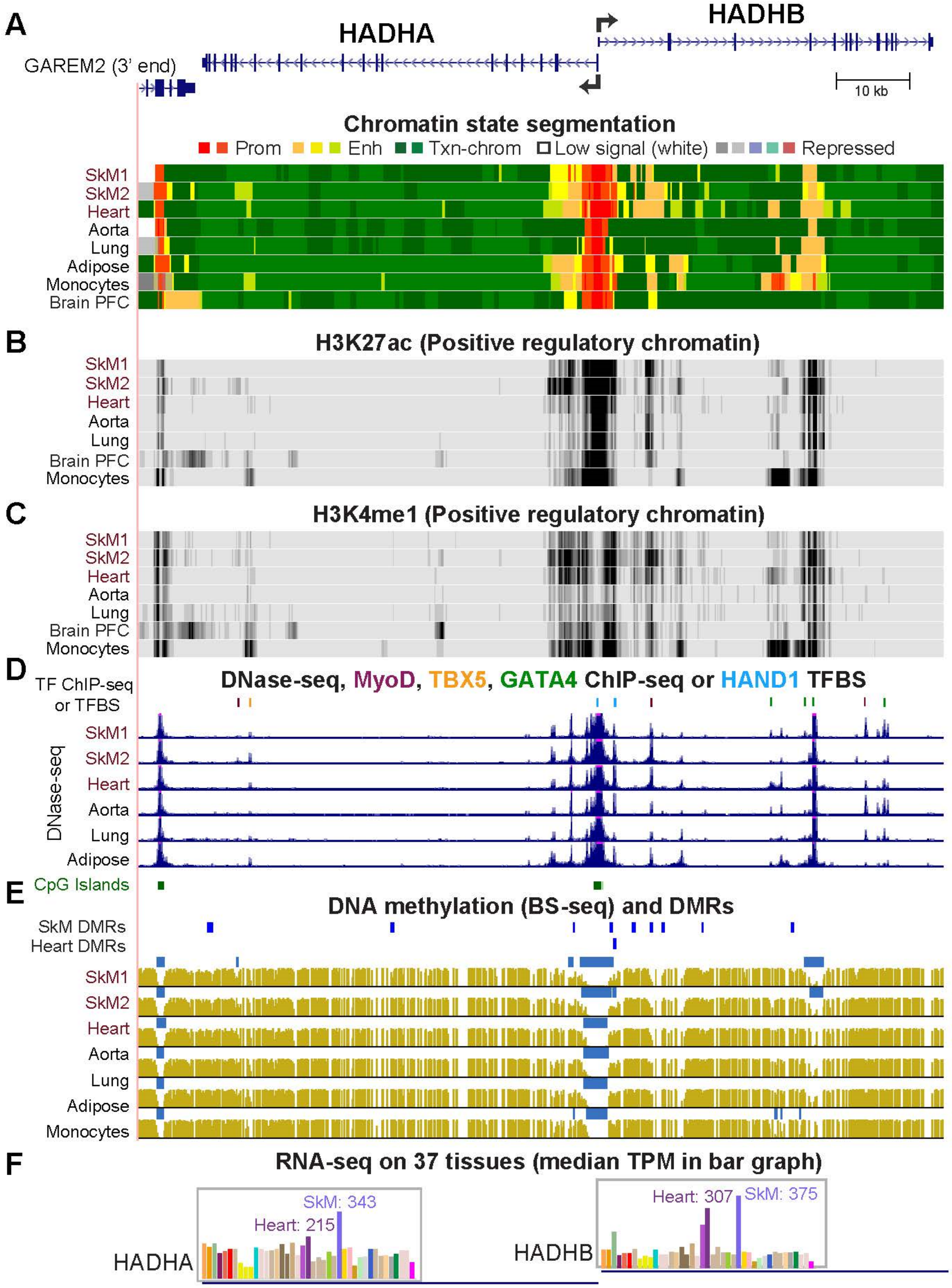

Figure S2. ***HADHB*** **and its co-expressed gene neighbor,** ***HADHA*****, share a bidirectional promoter with highest expression and DNA hypomethylation in skeletal muscle.** The indicated region for these two genes, which encode subunits of hydroxyacyl-CoA dehydrogenase, is chr2:26,404,644-26,514,796. **(A)** – **(C)** show chromatin state segmentation and ChIP-seq profiles for the two histone modifications that together indicate enhancer chromatin. **(D)** - **(F)** Similar to panels in Figure S1. Yellow segments in Panel **A**, weak enhancer chromatin; orange or yellow-green segments, strong enhancer chromatin.

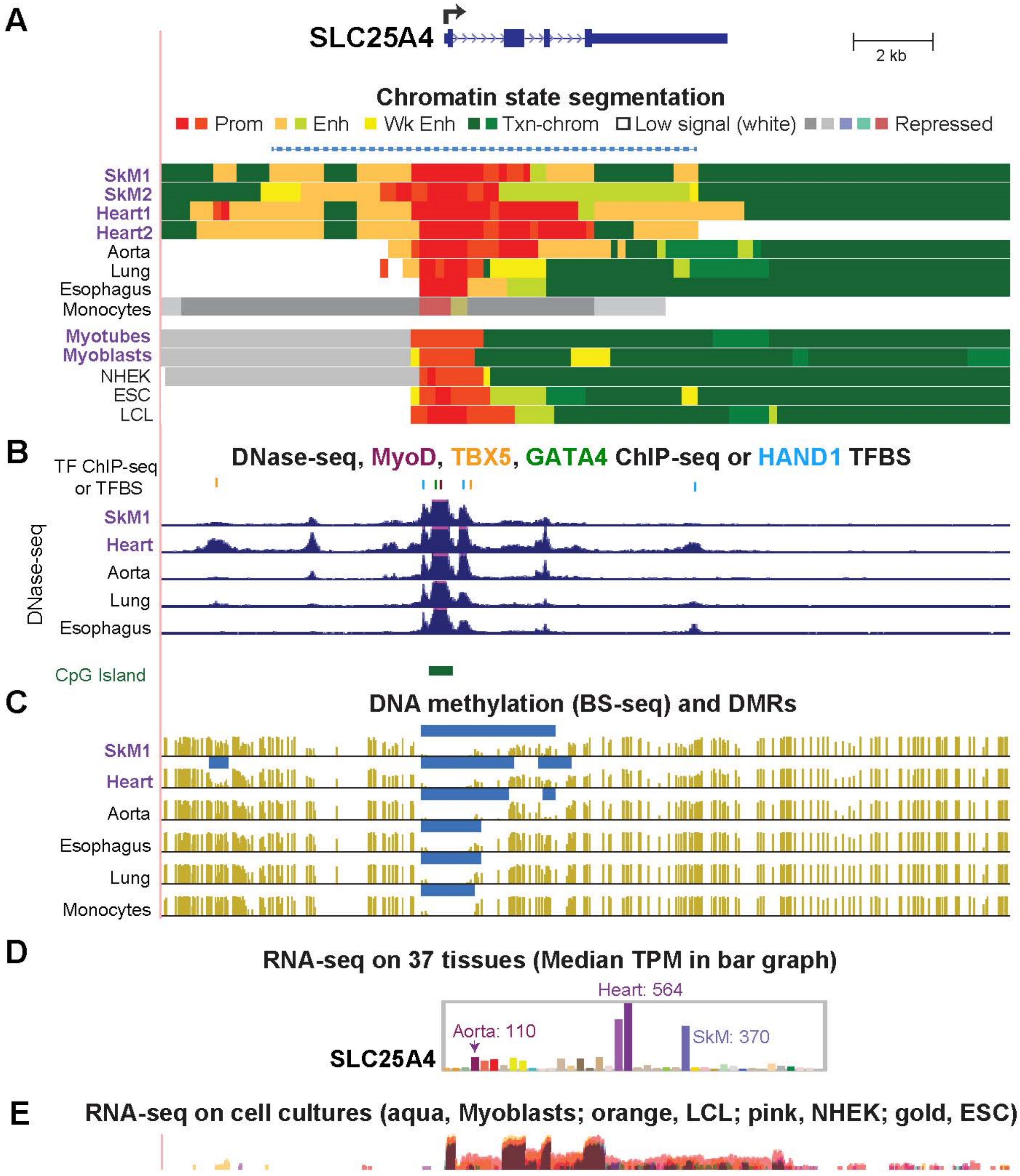

Figure S3. Preferential expression of *SLC25A4/ANT1* is correlated with an overlapping super-enhancer containing DNA hypomethylated subregions in skeletal muscle and heart. The region shown for this gene, which codes for a mitochondrial carrier adenine nucleotide translocator, is chr4:186,057,295-186,078,660. **(A)** – **(E)** are similar to panels in Figure S1. Super-enhancer chromatin (https://asntech.org/dbsuper/) in SkM and brain is indicated by a dotted light blue line over the top of the chromatin state tracks.

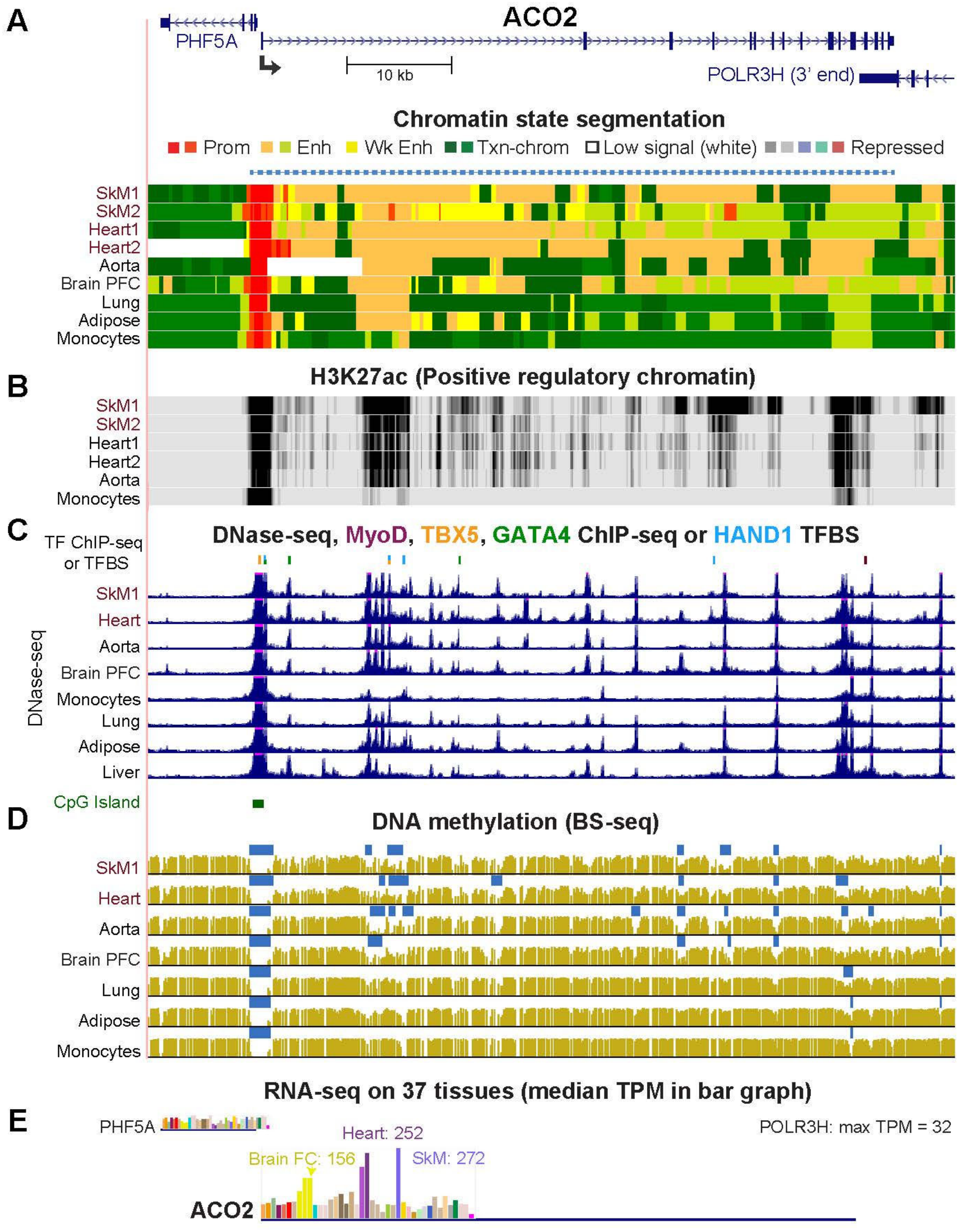

Figure S4. Preferential expression of *ACO2* is correlated with an overlapping a super-enhancer containing DNA hypomethylated subregions in SkM and heart. The region shown is chr22:41,854,342-41,930,734. (**A**) – (**E**) are similar to tracks in Figure S1. Super-enhancer is indicated by a dotted light blue line over the top of the chromatin state tracks.

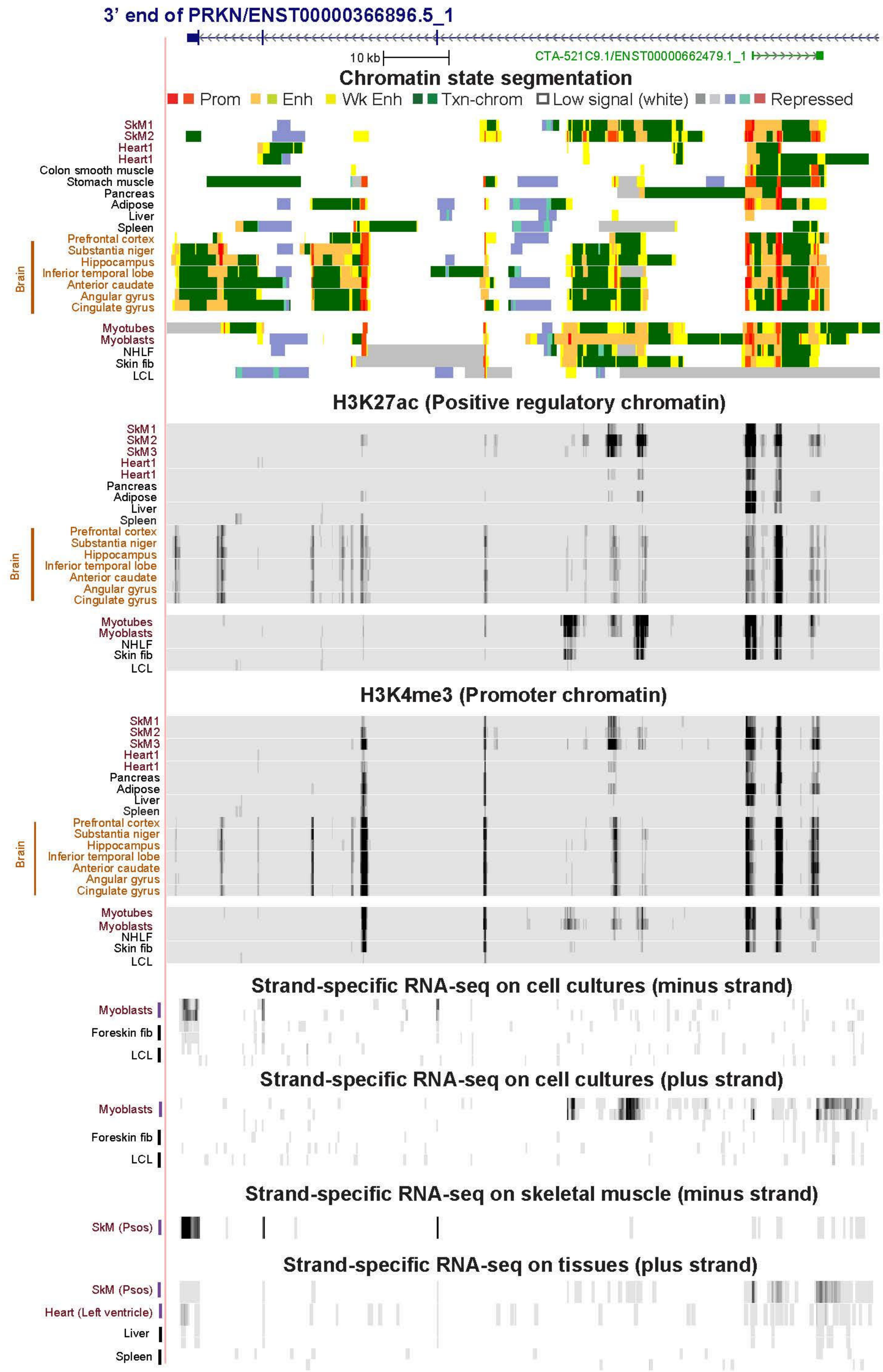

Figure S5. The myoblast-specific lncRNA from the 3' end of *PRKN* is an antisense transcript. The region shown is chr6:161,766,443-161,875,712. **(A)** Chromatin state segmentation tracks as in previous figures. **(B)** and **(C)** ChIP-seq profiles for H3K27ac and H3K4me3, the two modification that together signify promoter chromatin. **(D)** Strand-specific RNA-seq; vertical viewing range for Roadmap tissue samples, 0- 0.1 and for ENCODE RNA-seq samples, 0-15. Blue boxes, brain-specific epigenetic profiles; dotted black box, myoblast-specific epigenetic profiles or antisense RNA; purple box, SkM or myoblast-associated antisense RNA. Fib, fibroblasts

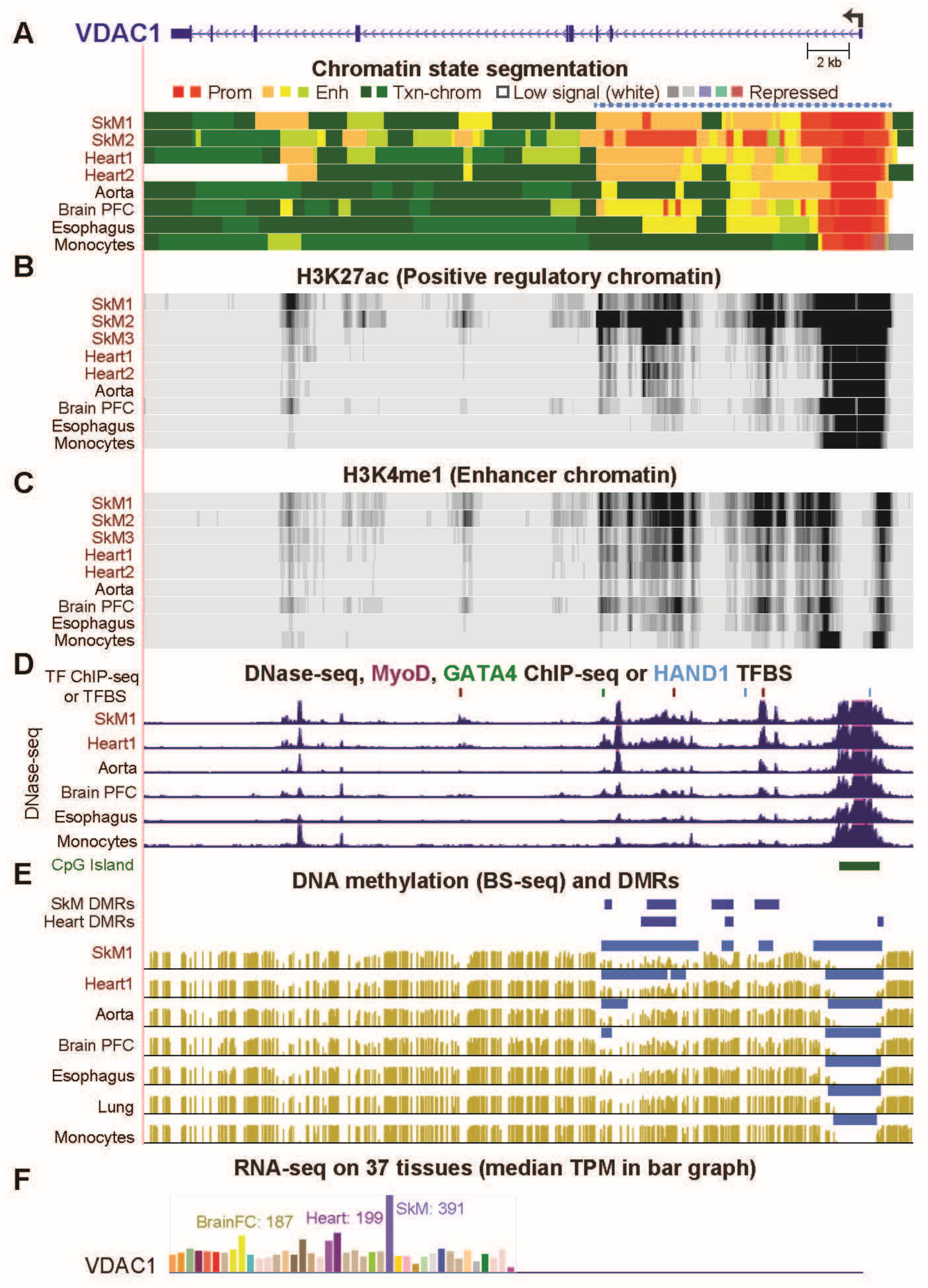

Figure S6. Preferential expression of *VDAC1* is correlated with an overlapping a super-enhancer containing DNA hypomethylated subregions in SkM and heart. The region shown is chr5:133,306,218-133,343,133. (**A**) – (**E**) are similar to panels in Figure S2.

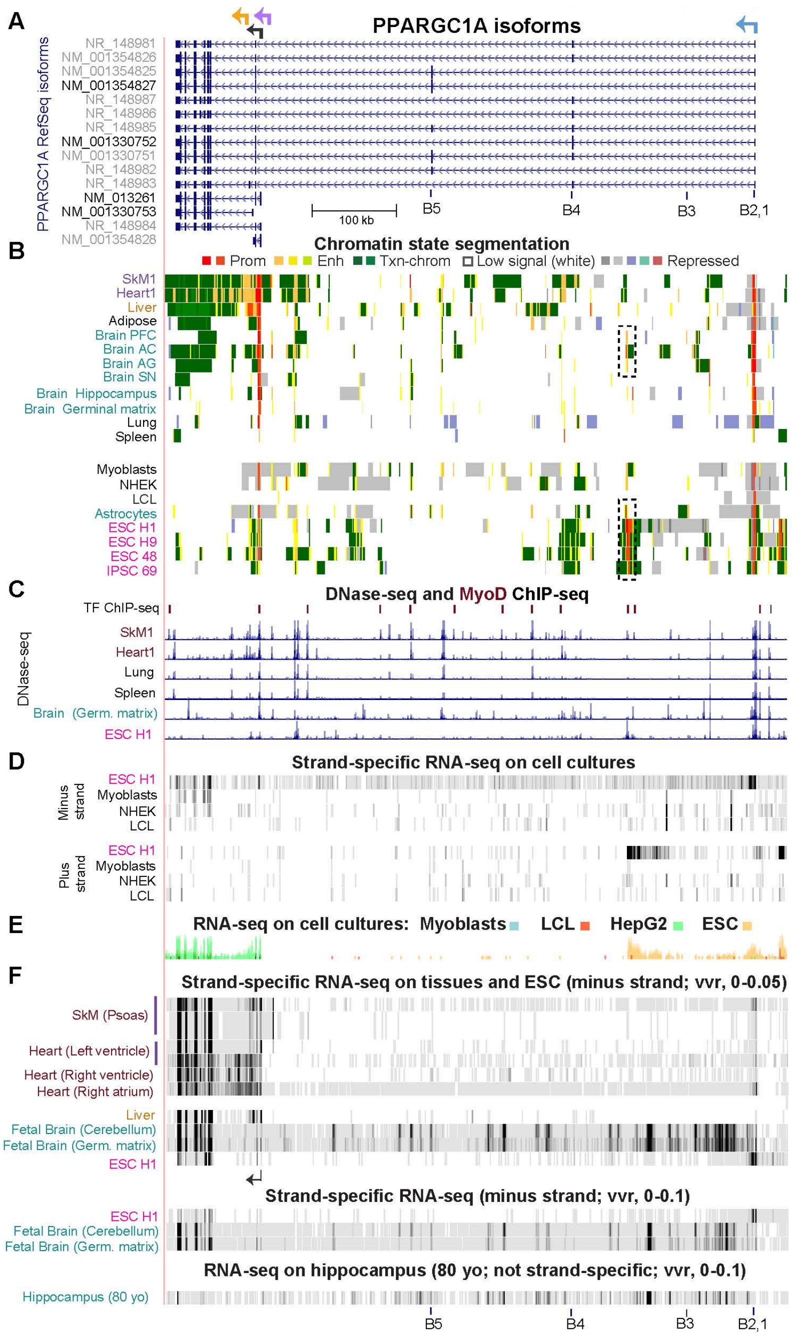

Figure S7. The full-length *PPARGC1A* gene including the far distal promoter displays novel brain and embryonal stem cell RNA signals. All RefSeq isoforms for *PPARGC1A* (chr4:23,780,312-24,511,140); missing from the RefSeq database is the Alt promoter isoform (Alt TSS, purple broken arrow). Full-length RefSeq isoforms other than the main described brain-associated isoforms [1] are shown in gray font. B1 – B5, previously described exons for brain-specific coding transcripts [1]; as in all figures, the taller boxes in the gene structures denote coding exons and shorter ones, non-coding exons. **(A)** – **(D)** and **(F)** are as in previous figures. Broken arrows in Panels **A** or **F,** TSS associated with the following alternative promoters: light blue, Distal; purple, Alt (Alternative); black, Prox (Proximal, the canonical promoter); orange, Liver. (**E**) Non-strand specific RNA-seq as an overlay graph; the RNA-seq signal at the canonical portion of *PPARGC1A* for ESC (see Panel **D**) is obscured by the higher HepG2 signal. Close-up of exons B4 and B5 showed that both cerebellum and germinal (Germ.) matrix have discrete RNA signal (not illustrated). Vvr, vertical viewing range for tissue RNA-seq; in Figure 7, the vvr for tissue RNA-seq was 0 – 0.1 Dotted boxes in Panel **B**, region of novel ESC intragenic AS promoter that align with an ESC-specific DHS in Panel **C**. ESC minus-strand RNA-seq repeated in Panel **F** for comparison to tissue RNA-seq. AC, anterior caudate; AG, angular gyrus; SN, substantia nigra.

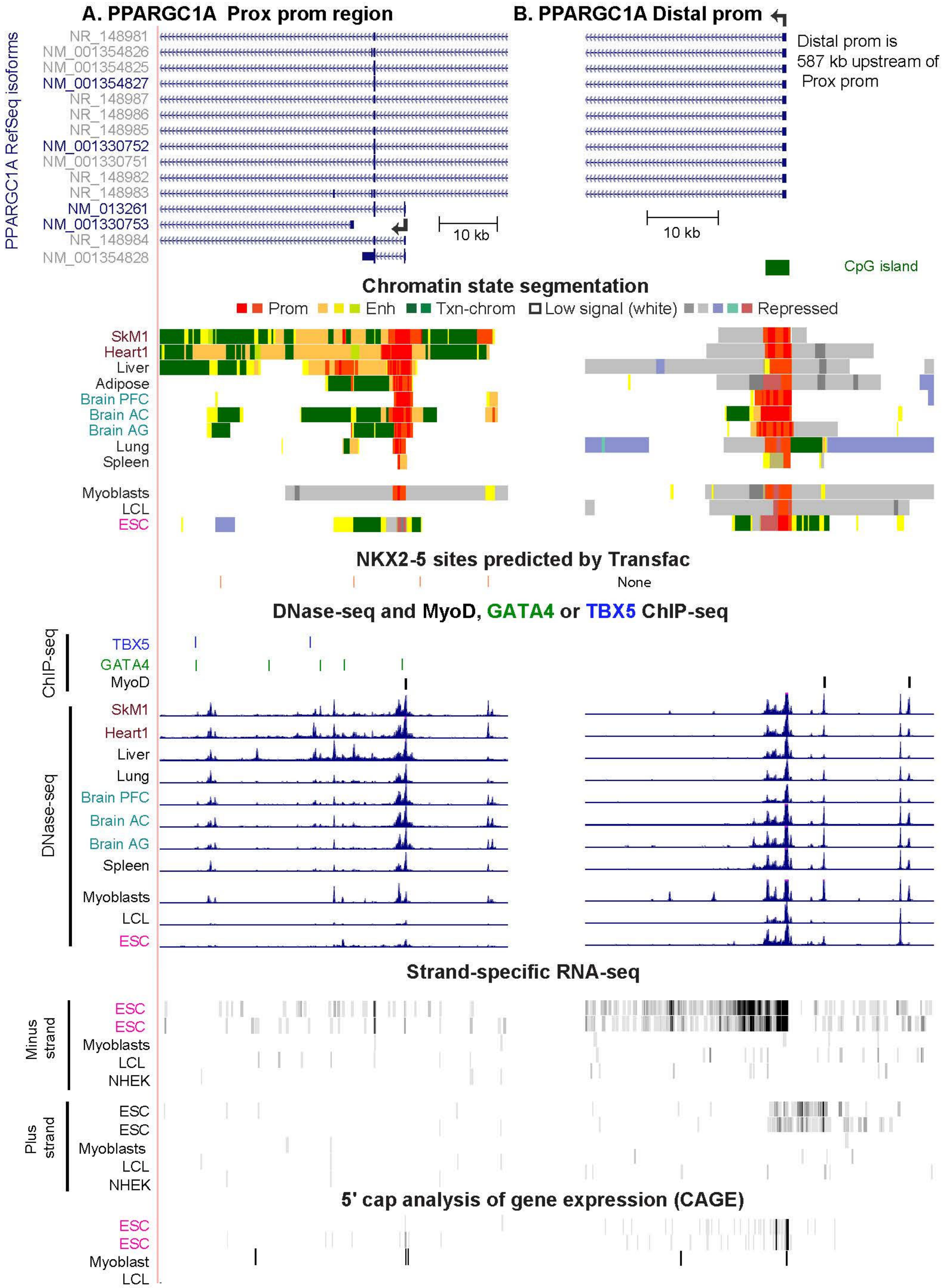

Figure S8. Closer view of the promoter regions of *PPARGC1A* shows tissue-specific epigenetic signatures. (A) The genomic region shown on the left is chr4:23,849,740-23,909,222 (59 kb). **(B)** The region shown on the right is chr4:24,446,483-24,495,085 (48 kb). Tracks are as in previous figures with the addition of 5′ cap analysis of gene expression for the 5′-ends of poly(A)$^+$ RNAs. The broken arrows for Liver, Prox, Alt, and Distal TSS are color coded as in Figure S7. Technical duplicates are shown for strand-specific ESC (H1) RNA-seq that indicate more signal at coding exon 2, which is the first exon common to *PPARGC1A* isoforms, than at exon 1 at the Prox TSS (Panel **A**) and yet more signal at non-coding exon B1 and downstream at the Distal TSS (Panel **B**). Only bivalent promoter chromatin was seen at the Prox promoter for ESC while strong promoter chromatin was found at the Distal promoter. The ESC-associated transcript is distinct from a previously described *PPARGC1A*-upstream EST from ESC and similar iPSC transcripts [2].

Reference

1. Soyal, S.M.; Felder, T.K.; Auer, S.; Hahne, P.; Oberkofler, H.; Witting, A.; Paulmichl, M.; Landwehrmeyer, G.B.; Weydt, P.; Patsch, W. A greatly extended PPARGC1A genomic locus encodes several new brain-specific isoforms and influences Huntington disease age of onset. *Hum Mol Genet* **2012**, *21*, 3461-3473, doi:10.1093/hmg/dds177.

2. Soyal, S.M.; Zara, G.; Ferger, B.; Felder, T.K.; Kwik, M.; Nofziger, C.; Dossena, S.; Schwienbacher, C.; Hicks, A.A.; Pramstaller, P.P., et al. The PPARGC1A locus and CNS-specific PGC-1α isoforms are associated with Parkinson's Disease. Neurobiol Dis 2019, 121, 34-46.
